# Development and trend of stem cell therapy for diabetes: a comprehensive review based on knowledge visualization

**DOI:** 10.3389/fimmu.2026.1856373

**Published:** 2026-07-08

**Authors:** Xinyu Huang, Qingguang Chen, Hao Lu

**Affiliations:** Department of Endocrinology, Shuguang Hospital Affiliated to Shanghai University of Traditional Chinese Medicine, Shanghai, China

**Keywords:** diabetes, stem cell therapy, immune modulation, bibliometric analysis, visualized knowledge mapping

## Abstract

**Introduction:**

Type 1 diabetes is characterized by autoimmune destruction of pancreatic β‑cells, whereas type 2 diabetes involves chronic low‑grade systemic inflammation. Stem cell therapy, through both direct cell replacement and immunomodulation, holds great promise for treating diabetes. However, a comprehensive bibliometric analysis systematically mapping the knowledge structure, evolutionary trajectory, and immunological landscape of this field is currently lacking.

**Methods:**

Using CiteSpace, VOSviewer, and the “bibliometrix” R package, we performed a comprehensive bibliometric analysis of 1,600 publications on stem cell therapy for diabetes retrieved from the Web of Science Core Collection (1995–2025). The analysis focused on the distribution of countries/regions, institutions, and authors, and examined research hotspots and development trends through co‑citation networks, keyword bursts, and cluster analysis.

**Results:**

The field has evolved through four developmental stages: concept validation (before 2007), clinical breakthrough of immune intervention (2007–2014), technological explosion of β‑cell differentiation (2014–2019), and optimization with complication‑oriented expansion (2019–present). Cell replacement and immunomodulation emerged as two core and increasingly prominent paradigms. Keyword burst analysis identified immunology‑related hotspots such as regulatory T cells, macrophage polarization, and exosomes. Mesenchymal stem cells and their exosomes exhibit great potential in immunomodulation and regeneration, opening broad prospects for the treatment of diabetes and its complications.

**Discussion:**

The field is moving toward more refined cell engineering, with notable progress in understanding the mechanisms of β‑cell maturation and differentiation. Future research will place greater emphasis on the development of immune evasion technologies, including the design of novel biocompatible encapsulation materials and the exploration of local immunomodulatory strategies. This will require deep integration of cell biology, immunology, genetic engineering, and clinical medicine to address fundamental issues related to cell function, immune rejection, and long‑term safety, thereby providing safer, more effective, and more universally applicable therapeutic strategies for diabetes.

## 1 Introduction

Diabetes mellitus is a chronic metabolic disorder characterized by hyperglycemia. It results from either insulin secretion defects or insulin resistance, which lead to prolonged elevated blood glucose levels and associated biochemical abnormalities. This, in turn, causes chronic damage and functional impairment to target organs and tissues such as the heart, blood vessels, kidneys, and nerves. The incidence of diabetes has been increasing annually, becoming a global health challenge. Epidemiological studies indicate that, in 2021, diabetes affected 529 million people, with projections suggesting that by 2050, the number of affected individuals will rise to 1.31 billion (1). Due to the stringent dietary restrictions and lifestyle management required for diabetes, coupled with the potential risk of hypoglycemia associated with various glucose-lowering medications, achieving and maintaining optimal glycemic control remains a significant challenge for both patients and healthcare providers.

Pancreatic islet transplantation is one of the most promising treatments for diabetes, as it can replace damaged pancreatic β-cells and enable glucose-responsive insulin secretion. Numerous studies have demonstrated that islet transplantation can reduce or even eliminate the need for exogenous insulin in diabetic patients (2). However, the widespread application of this therapy is constrained by three major limitations: a severe shortage of donor pancreata, islet damage during isolation and transplantation, and the requirement for lifelong immunosuppressive therapy to prevent rejection (3, 4). Collectively, these obstacles make the restoration of pancreatic endocrine function a critical unmet clinical challenge. In this context, stem cell therapy—with its unique regenerative and immunomodulatory properties—has emerged as a promising alternative to overcome these barriers and achieve functional cure for diabetes.

Stem cells are asexual lineage cells characterized by their capacity for self-renewal and differentiation into multiple functional somatic cell types. Based on their origin, developmental stage, and potency, stem cells can be broadly classified into three categories: adult stem cells, embryonic stem cells(ESCs), and induced pluripotent stem cells (iPSCs). It is noteworthy that ESCs are derived from early-stage embryos, which raises ethical concerns, whereas adult stem cells and iPSCs generally do not present such ethical issues (5).

Due to their potential regenerative capabilities, stem cells are often referred to as “universal cells” in the medical field. The external regulation of stem cell fate has enabled more precise control over their self-renewal and differentiation processes (6–8). A study published in Signal Transduction and Targeted Therapy reported that globally, 1,426 stem cell clinical trials are ongoing or completed, covering more than 20 major categories of systemic diseases. Some of these trials have already demonstrated clear therapeutic effects and promising prospects (9). Currently, the application of stem cells in the treatment of diabetes and its complications mainly focuses on two aspects: First, utilizing the differentiation ability of stem cells to cultivate functional islet cells, which can secrete insulin through transplantation to achieve cell organ replacement. Second, transplanting mesenchymal stem cells(MSCs) to regulate immunity, protect undamaged β-cells, and improve the pancreatic environment, thereby alleviating diabetes symptoms (10). In past research, significant progress has been made in the technology of differentiating human pluripotent stem cells (hPSCs) into β-cells. β-like cells derived from hPSCs have achieved functional activity both *in vitro* and *in vivo*, marking a major technological breakthrough as they gradually resemble authentic human islets (11–13). Since the concept of “adult mesenchymal stem cells” was introduced in 1991, a series of identification criteria for MSCs have been subsequently proposed (14). The discovery of MSCs has initiated a new era in preclinical research and clinical trials. These studies have evaluated the safety and efficacy of MSCs in treating various diseases and have facilitated their translation from basic research to clinical applications. MSCs have emerged as a promising option for improving diabetes symptoms and controlling blood glucose, owing to their wide availability, robust self-renewal capacity, multipotent differentiation, low immunogenicity, immunomodulatory properties, and anti-apoptotic effects (9). Although the role of stem cell therapy in diabetes was once controversial, its practical value has become increasingly recognized, and the therapy is now being translated into clinical practice. Nevertheless, a comprehensive and systematic analysis of the research landscape—including macro-level trends, hotspots, and developmental trajectories—remains lacking. Therefore, this study aims to conduct a longitudinal bibliometric analysis of relevant articles over recent decades and to visually map the knowledge structure of stem cell therapy in diabetes. This approach will provide researchers with a broader and deeper understanding of the field, offering valuable references for future investigations and inspiring new research directions.

Bibliometrics, as an emerging research method, utilizes mathematical and statistical techniques to conduct comprehensive quantitative and qualitative reviews and analyses of literature. By performing multi-dimensional dynamic analysis of factors such as countries, authors, institutions, journals, keywords, and references within a specific research field, bibliometrics provides detailed information on the research development status, distribution patterns, research hotspots, and trends in that field. This method holds reliable value for evaluating the research trends and hotspots of stem cell therapy in the application to diabetes. In this study, we focus on biomedical research related to the application of stem cells in diabetes and its complications, aiming to provide a comprehensive overview of the current research landscape in this field. To achieve this, we first performed a quantitative analysis of the growth trends of stem cell therapy–related publications and identified the key contributors, including major countries, authors, and institutions involved. Next, based on keyword co–occurrence mapping, we conducted a detailed discussion of the research hotspots, with an emphasis on the two major paradigms of stem cell therapy for diabetes — cell replacement and immunomodulation — and summarized the key immunological parameters reported in representative clinical and preclinical studies. Finally, we outlined the current shortcomings and challenges of stem cell–based therapy for diabetes and proposed future research directions.

## 2 Materials and methods

### 2.1 Data collection and search strategy

**Figure 1:**
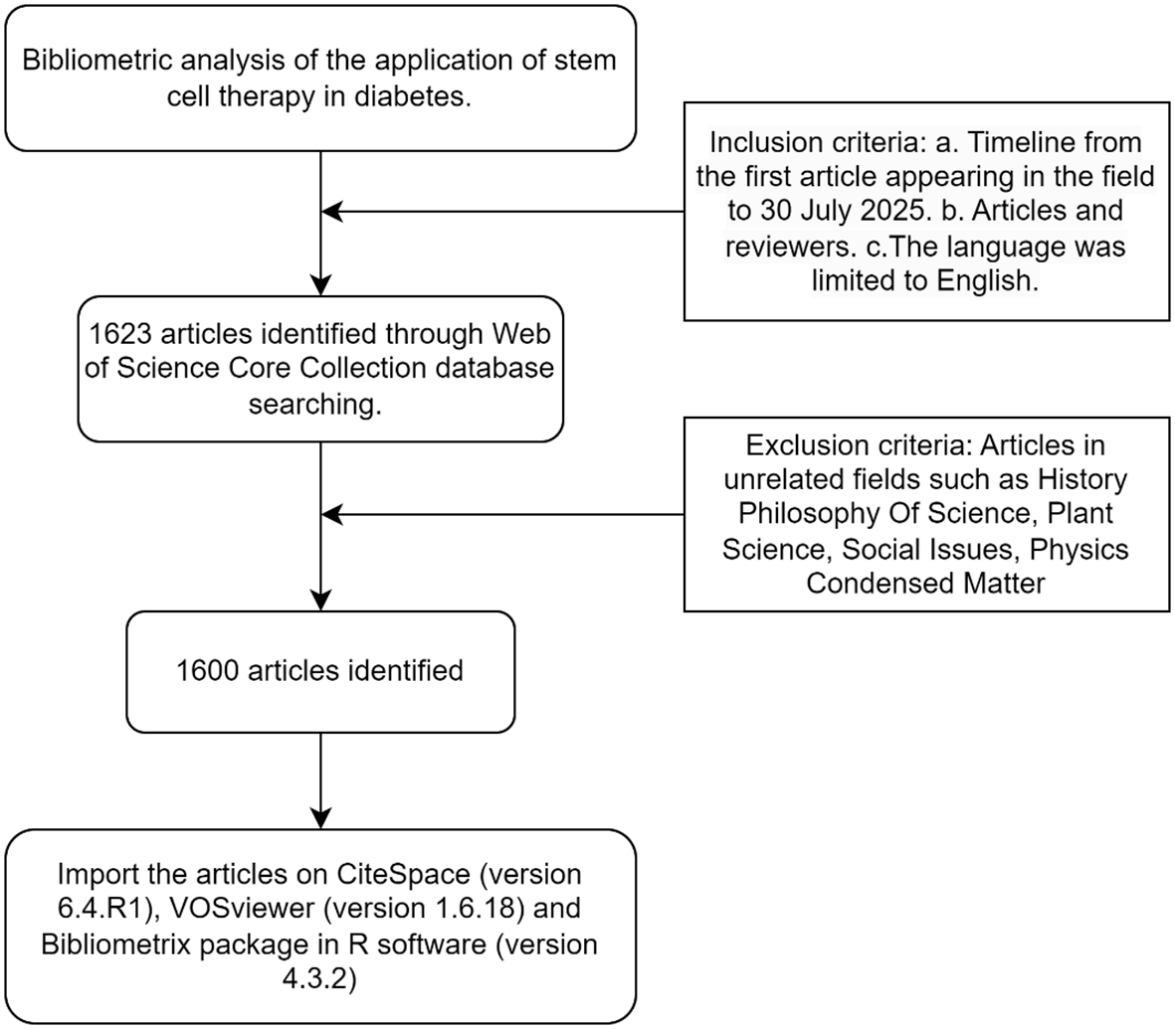
Flowchart of the literature search and screening procedures used in the study.

The data for this study were obtained from the Web of Science Core Collection, covering publications from the database inception to July 30, 2025. Only English-language articles were included, and the document types were limited to “review” and “article. The search strategy was set as follows:

(((diabet* OR “type 1 diabet*” OR T1D OR “type 2 diabet*” OR T2D OR GDM) AND (“stem cell therap*” OR “stem cell transplant*” OR “stem cell differentiat*” OR “iPSC derived beta cell*” OR “MSC immunomodulation” OR “pluripotent stem cell* engineer*” OR “progenitor cell replacement”)) OR ((“embryonic stem cell” OR ESC) AND (“diabet* treatment” OR “beta cell generation”)) OR ((“islet-like cell*” OR “insulin-secreting cell*”) AND (“stem cell origin” OR “iPSC differentiat*”))). A total of 1,623 articles were retrieved. After manual screening, irrelevant disciplines (e.g., History & Philosophy of Science, Plant Science, Social Issues, Physics Condensed Matter, Dentistry Oral Surgery Medicine, Veterinary Science) were excluded to prevent the co–occurrence network from deviating from the core theme. Ultimately, 1,600 articles were retained. The literature screening flow chart is shown in **Figure 1**.

### 2.2 Data preprocessing and deduplication

The retrieved records were exported as text files in “Full Record and Cited References” format and named “download_1–500.txt”, “download_501–1000.txt”, “download_1001–1500.txt”, and “download_1501–1600.txt”. Disambiguation was performed for countries, authors, and institutions, and keywords were standardized by merging synonyms and unifying singular/plural forms.

### 2.3 Data analysis and visualization

The analytical functions of the Web of Science database were used to summarize the annual numbers of publications and citations on stem cell applications in diabetes. The data were then imported into CiteSpace (version 6.4.R1), VOSviewer (version 1.6.18), and the “bibliometrix” R package (R version 4.3.2) for visualization analysis and knowledge mapping of countries, authors, institutions, references, keywords, etc.

#### 2.3.1 CiteSpace 6.4.R1 settings

Time slicing: 1995–2025, with years per slice = 1. Node types: country, institution, author, keyword, and reference (selected sequentially). Selection criteria: Top N = 50 per slice. Pruning: Pathfinder + Pruning sliced networks (to simplify the network while preserving key structures). Clustering algorithm: Log-likelihood ratio (LLR) for keyword cluster labeling.

#### 2.3.2 VOSviewer 1.6.18 settings

Node types: authors, keywords, co-cited journals, and co-cited references. Counting method: full counting. Normalization: association strength. Layout: gravity algorithm (attraction = 2, repulsion = –1). Clustering resolution: default 1.0.

#### 2.3.3 “Bibliometrix” R package (R version 4.3.2) settings

Used for: Annual publication trend analysis. Core journal identification (Bradford’s law). Country collaboration network visualization.

## 3 Results

### 3.1 Annual publication and citation trends

**Figure 2:**
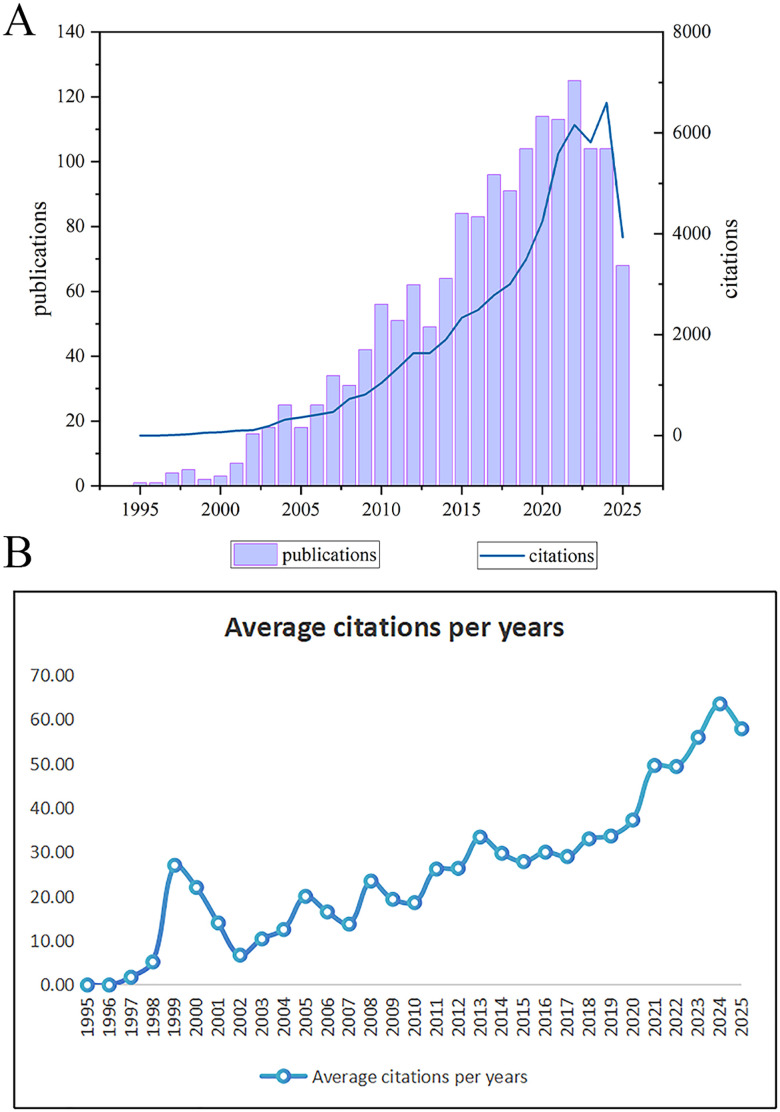
Publication output and the literature citations. **(A)**The combination chart presents the annual publication count and average citation per year of stem cell therapy for diabetes from 1995-2025; **(B)** Figure B displays the average annual citation count per publication.

From 1995 to 2025, a total of 1,600 articles related to the application of stem cell therapy in diabetes have been published. **Figure 2A** presents the annual distribution of publication volume and citation frequency in this research field from 1995 to 2025. These two indicators are core metrics in bibliometrics used to assess the scientific output and academic impact of a discipline. According to the bar chart in **Figure 2A**, the highest annual publication volume occurred in 2022, with 125 articles published, indicating that research activities in this field were most active in 2022, reaching a peak in output. The line chart suggests that the peak in annual citation frequency occurred in 2024, with 6,595 citations, which aligns with the general pattern in literature research where citation frequency typically lags behind publication volume. This is because newly published papers require time to be read, absorbed, and cited by subsequent research. The citation peak in 2024 directly reflects the academic influence generated by the publication peak in 2022. **Figure 2B** presents the average number of citations for annual publications.

The publication trend in this field can be revealed through three stages, reflecting the lifecycle of its development from infancy, acceleration, to explosive growth: a. From 1995 to 2001, both the number of publications and citation frequency in this field were at very low levels, with the annual publication volume not exceeding 10 papers. b. From 2002 to 2009, the field experienced relatively rapid development compared to the previous period, with both the number of publications and citations increasing accordingly. However, the annual publication volume still did not exceed 50 papers. c. Since 2010, there has been a significant increase in the number of publications, peaking historically in 2022. Although data from 2023 shows a slight decline, this is typically due to delays in database indexing rather than an actual reduction. This trend graph of publication volume and citation frequency reveals the entire process of stem cell therapy in diabetes applications evolving from an emerging interdisciplinary field to a cutting-edge, popular domain. It also suggests that this field may be in the “pre-maturity” phase of its scientific evolution cycle, and we predict that the number of publications in this field will continue to grow in the future.

### 3.2 Distribution of countries/regions and institutions

**Figure 3:**
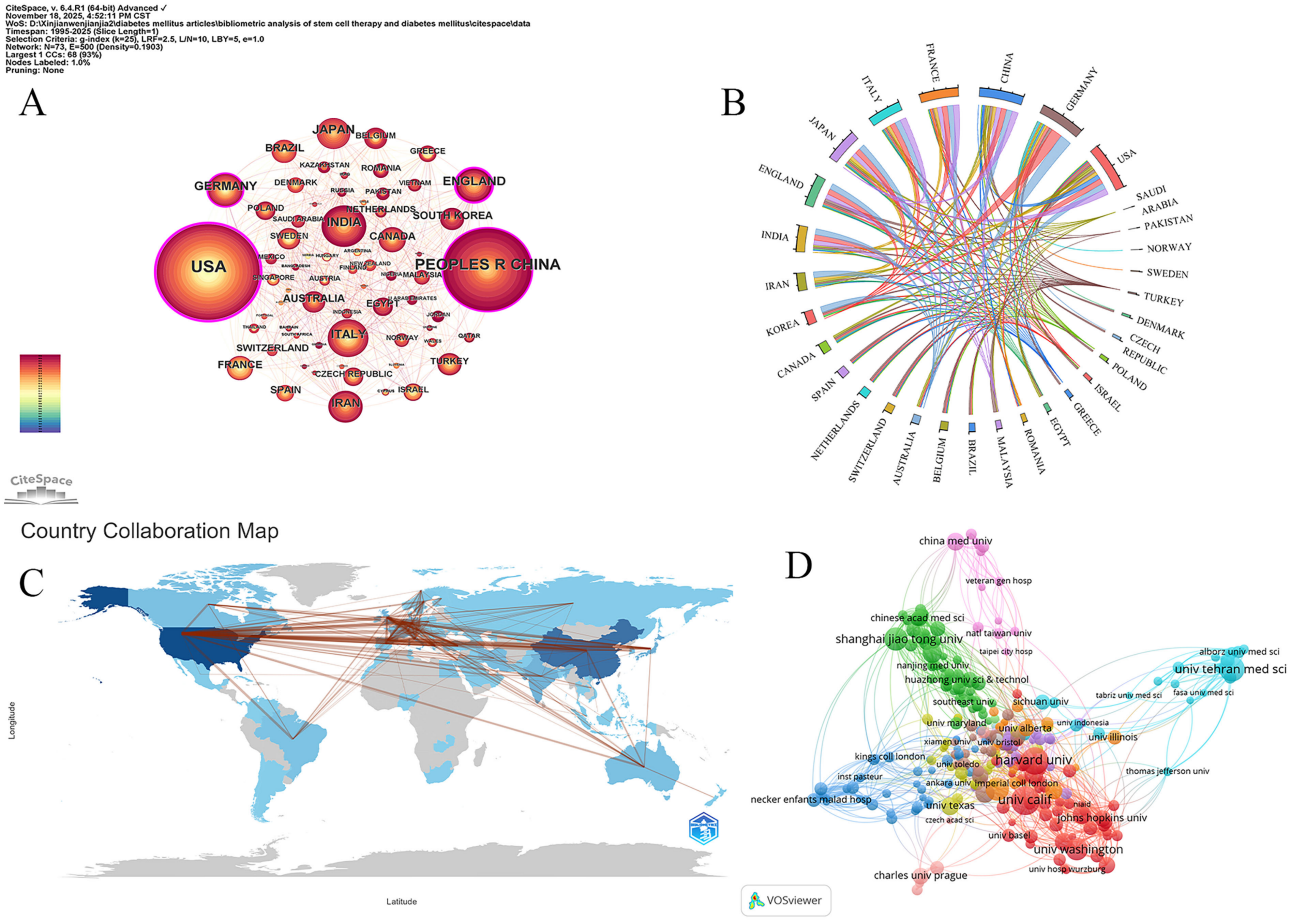
Cooperation map of countries/regions and institutions in stem cell therapy for diabetes. **(A)** A visual map for the CiteSpace network among countries. **(B)** Network map for scientific cooperation among countries/regions. **(C)** A visual map for the international country collaboration. **(D)** A visual map for the VOSviewer network among institutions.

**Figure 3A** presents the national co-occurrence map of stem cell therapy and diabetes research, while **Table 1** shows the top ten countries/regions with the highest number of published papers in this field. The country with the most publications is the United States (504 papers), followed by China (351 papers), the United Kingdom (106 papers), and Japan (103 papers). The United States and China account for 31.5% and 21.94% of the total publications, respectively, together making up more than 50% of the total output. This indicates a clear “bipolar dominance with multipolar follow-up” pattern in the distribution of national research output in this field, and highlights the uneven international distribution of research in this area. According to the citation counts in **Table 1**, the United States and China remain the most frequently cited countries. However, it is noteworthy that the total citation count of the United States (27,297 citations) is more than three times that of China (8,809 citations), suggesting that American academic achievements are more widely referenced and cited, demonstrating its substantial cumulative academic influence and global leadership. In terms of average citations per paper, France (107.71), the United Kingdom (62.93), Italy (62.64), and Germany (55.73) rank at the top, indicating that the papers published by these countries are of high quality and have significant influence in this field. **Figures 3B, C** illustrate the collaboration networks among countries in this field, with the United States, Germany, and China showing strong collaborative relationships.

**Table 1:** Top 10 most productive countries/regions in GM and hypertension.

Rank	Countries/regions	Record count	Percentage (%)	Average per item	Citations	Total link strength	Centrality
1	USA	504	31.50	54.16	27297	298	0.43
2	CHINA	351	21.94	25.10	8809	100	0.17
3	ENGLAND	106	6.63	62.93	6671	189	0.26
4	JAPAN	103	6.44	32.18	3315	78	0.08
5	INDIA	98	6.13	38.46	3769	55	0.04
6	ITALY	90	5.63	62.64	5638	136	0.02
7	GERMANY	77	4.81	55.73	4291	135	0.12
8	IRAN	62	3.88	23.42	1452	35	0.07
9	FRANCE	49	3.06	107.71	5278	106	0.02
10	AUSTRALIA	48	3.00	41.63	1998	58	0.05

**Figure 3D** presents the co-occurrence map of publishing institutions in this field. **Table 2** displays the top ten institutions with the highest number of publications. The University of California System (42 papers), Harvard University (38 papers), and Tehran University of Medical Sciences (33 papers) occupy the top three positions. Notably, six of the top ten institutions are based in the United States, and all of these institutions have a high cumulative citation count. Although the University of Florida published only 17 papers, it ranks first in total citations with 2,955 citations. This further reinforces the leading position of the United States in this field. It also highlights that top-tier research universities in the U.S. are the primary sources of scientific output in this area, serving as the global driving force behind stem cell therapy research for diabetes. These institutions not only lead in research output but also play a dominant role in research quality and international collaboration. Additionally, developing countries such as Iran, China, and Brazil are beginning to emerge as key players in this field, indicating that the research landscape is evolving towards greater diversity and globalization.

**Table 2:** Top 10 institutions based on publications.

Rank	Organization	Documents	Citations	Total link strength	Centrality
1	University of California System	42	2495	67	0.19
2	Harvard University	38	2143	82	0.11
3	Tehran University of Medical Sciences	33	658	74	0.04
4	Shanghai Jiao Tong University	27	801	61	0.02
5	University of São Paulo	27	1252	35	0.03
6	University of Washington	25	1910	58	0.04
7	Nanjing University	19	439	29	0.01
8	Miami University	19	662	30	0.01
9	Stanford University	17	1331	17	0.07
10	University of Florida	17	2955	10	0.05

### 3.3 Analysis of authors and co-cited authors

An analysis of the authors of published papers reveals the key scholars and core research forces within the field. The **Table 3** shows the top ten authors ranked by publication count, co-citation frequency, affiliated institutions, and total link strength. According to Price’s Law, the minimum publication count for a core author in a research field is given by m = 0.749 √nmax () (where nmax is the maximum number of publications by any author)≈ 2.9. Therefore, authors with three or more publications (inclusive) are considered core authors in the field. A total of 271 authors meet this threshold, indicating that the field of stem cell research for diabetes has established a stable and strong research base. The authors with the highest number of publications in this field are Julio Cesar Voltarelli from the University of São Paulo and Bagher Larijani from the Endocrinology and Metabolism Research Center at Tehran University of Medical Sciences, both of whom have published 15 papers. Following them are Susumu Ikehara from Kansai Medical University with 13 papers, and Carlos E. B. Couri (11 papers) and Maria Carolina Oliveira (11 papers), both from the University of São Paulo. The author with the highest number of citations and co-citations in this field is also Julio Cesar Voltarelli, with a total of 1,041 citations. This strongly demonstrates his significant impact on the field, contributing to the University of São Paulo’s high publication output and citation count. Notably, five of the top ten authors by publication count are from the University of São Paulo, with representative figures being Voltarelli and Couri. All five authors have high citation counts and total link strength, which suggests that this group forms a highly influential and collaborative core team in the field of stem cell therapy for diabetes.

**Table 3:** Top 10 authors in stem cell therapy for diabetes.

Rank	Author	Record count	% of 1600	Citations	Average per item	H-index	Affiliations	Total link strength
1	Voltarelli, Julio Cesar	15	0.94	1041	69.40	12	University of São Paulo	73
2	Larijani, Bagher	15	0.94	143	9.53	6	Tehran University of Medical Sciences	40
3	Ikehara, Susumu	13	0.81	478	36.77	11	Kansai Medical University	21
4	Couri, Carlos E B	11	0.69	942	85.64	12	University of São Paulo	67
5	Oliveira, Maria Carolina	11	0.69	950	86.36	9	University of São Paulo	70
6	Simoes, Belinda Pinto	10	0.63	931	93.10	9	University of São Paulo	70
7	Ricordi, Camillo	10	0.63	460	46.00	10	University of Miami	22
8	Moraes, Daniela Aparecida	9	0.56	899	99.89	8	University of São Paulo	69
9	Bhonde, Ramesh Ramchandra	9	0.56	224	24.89	6	Manipal University	9
10	Dominguez-bendala, Juan	9	0.56	343	38.11	7	University of Miami	16

**Figure 4:**
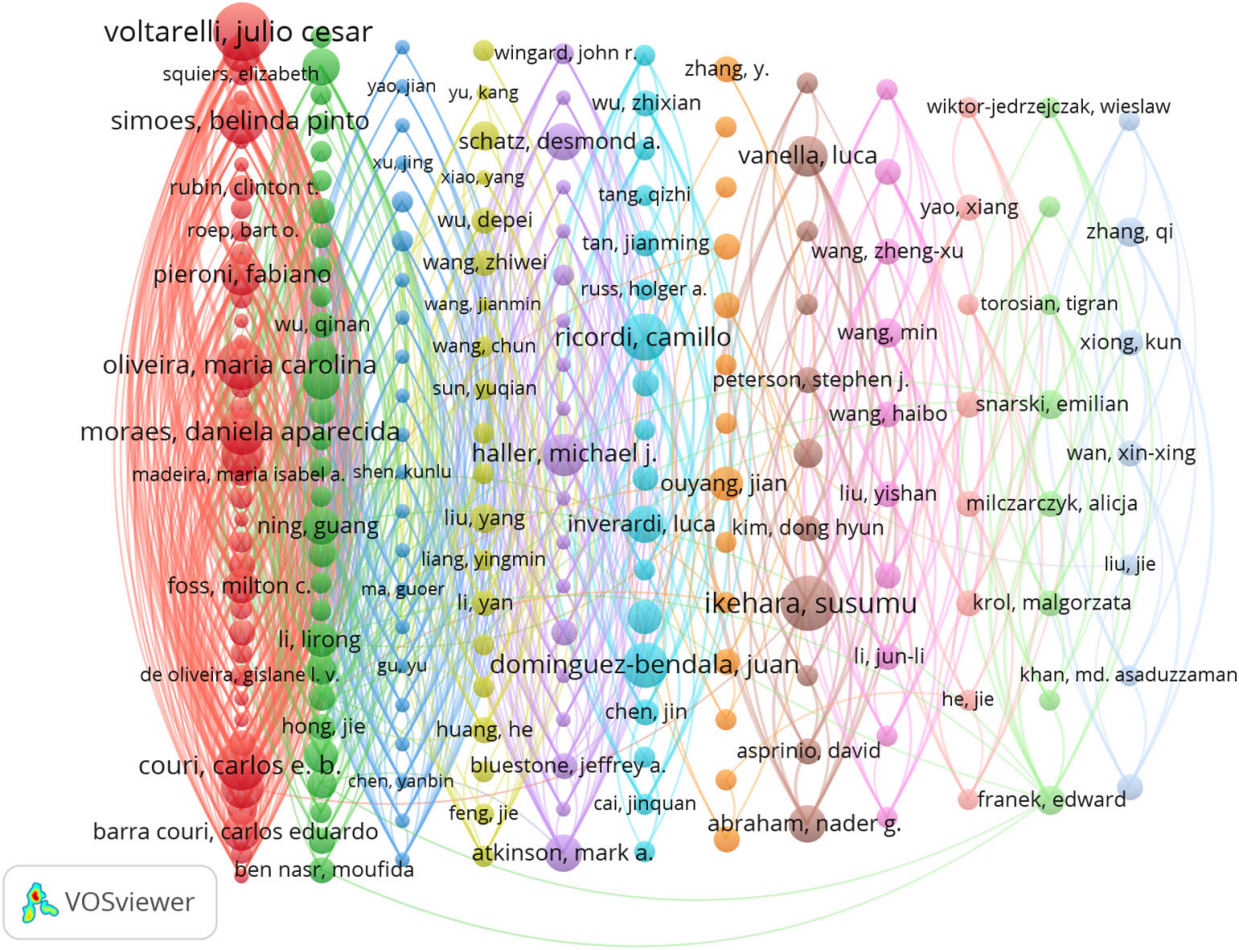
A visual representation of the co-authors’ collaborative network. Different colored clusters represent distinct author categories.

The visual map of authors in this field is presented through VOSviewer and Pajek (**Figure 4**). The size of each node represents the author’s publication output, with each color indicating a different author group. The lines connecting the nodes represent collaborative relationships between authors, with thicker lines indicating stronger collaboration. The map displays the 12 largest clusters in the field, each of which is interconnected, suggesting that the research among these scholars is related. The red cluster primarily consists of researchers from the University of São Paulo, including figures like Julio Cesar Voltarelli and Carlos E. B. Couri. This team focuses on the potential applications of stem cell therapy and immune interventions in type 1 diabetes(T1DM), with particular emphasis on the preservation and regeneration of β-cell function.

The team, led by key scholars from Tehran University of Medical Sciences, including Bagher Larijani, Ali Tootee, and Ensieh Nasli Esfahani, primarily focuses on stem cell therapy, particularly the potential of MSCs and hematopoietic stem cells for treating T1DM. Their research emphasizes the underlying immunological mechanisms and clinical efficacy of these therapeutic approaches. The brown cluster consists of a research team led by Susumu Ikehara from Kansai Medical University. This team primarily focuses on stem cell transplantation, including various approaches such as IBM-BMT, combined thymus transplantation, and HO-1 induction, to regulate immune imbalance, improve insulin sensitivity, and promote the restoration of β-cell function. The blue cluster is centered around a team of researchers, including Camillo Ricordi and Juan Dominguez-Bendala. Their work emphasizes addressing the issues of insufficient pancreatic islet cell sources and immune rejection through multidisciplinary techniques, including transcription factor regulation, cell reprogramming, MSCs differentiation, and encapsulation preservation. Their primary focus is on pancreatic function reconstruction and the potential for replacing exogenous insulin therapy. The research team led by Ramesh Ramchandra Bhonde concentrates on exploring the differentiation potential of MSCs from various sources, such as dental pulp, placenta, and hair follicles, into insulin-producing cells (IPCs) and their application in diabetes treatment. The aim is to efficiently generate functional IPCs by optimizing differentiation protocols and discovering new stem cell sources. The purple cluster is led by core researchers Michael J. Haller, Desmond A. Schatz, and Mark A. Atkinson. Their research focuses on exploring the immunomodulatory and therapeutic potential of umbilical cord blood cell therapies, including whole blood transfusion and expanded regulatory T cells, in T1DM. Additionally, their work addresses the safety of these treatments, the challenges that need to be resolved in future clinical trials, and the mechanisms and long-term efficacy of such therapies.

### 3.4 Analysis of co-cited references

**Figure 5:**
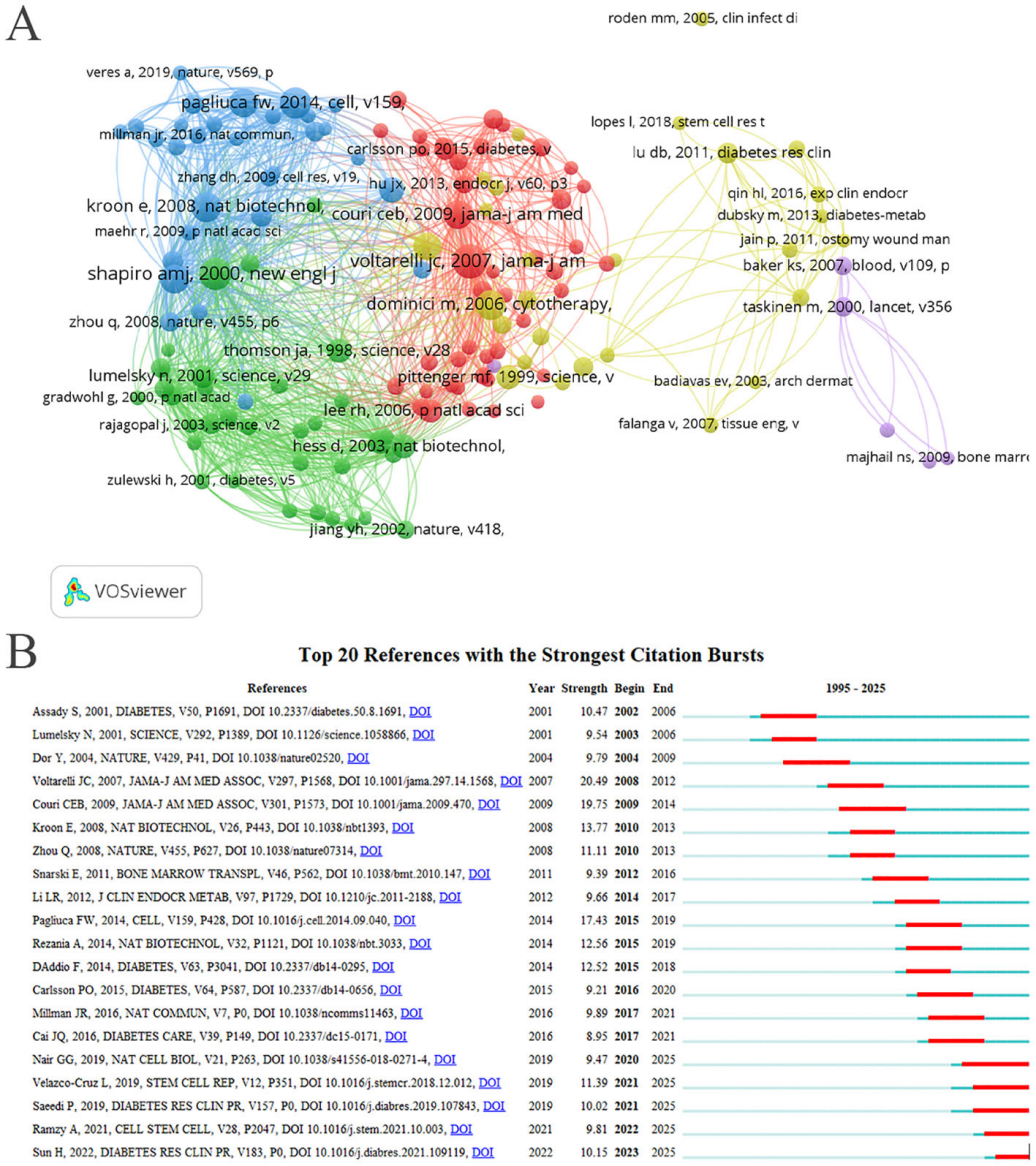
The visual analysis of highly cited references related to stem cell therapy for diabetes. **(A)** References co-citation network in stem cell therapy for diabetes. **(B)** Top 20 references with the strongest citation bursts in stem cell therapy for diabetes.

Co–citation analysis was employed to identify the intellectual base of the field. Co–citation refers to the frequency with which two documents are simultaneously cited by a third document, and this frequency indicates the strength of the thematic relationship between the two documents. **Table 4** presents the top ten most co-cited papers, with the most cited being ‘C-Peptide Levels and Insulin Independence Following Autologous Nonmyeloablative Hematopoietic Stem Cell Transplantation in Newly Diagnosed Type 1 Diabetes Mellitus’ by Carlos E. B. Couri, published in JAMA (43 citations). The second most cited paper is also published in JAMA and written by Júlio C. Voltarelli, titled ‘Autologous Nonmyeloablative Hematopoietic Stem Cell Transplantation in Newly Diagnosed Type 1 Diabetes Mellitus’ (42 citations). Notably, these two articles were authored by Carlos E. B. Couri and Júlio C. Voltarelli, who have a close collaborative relationship. The third most co-cited paper is ‘Generation of Functional Human Pancreatic β Cells *In Vitro*’ by Felicia W. Pagliuca, published in Cell (34 citations). Among the top 10 most co-cited papers, 6 are basic experimental studies, 3 are clinical trial studies, and 1 is an epidemiological survey report. **Figure 5A** displays the co-citation network, and based on this co-citation map, we further performed a co-citation burst analysis. Citation burst refers to a phenomenon where a paper’s citation frequency significantly exceeds its historical baseline level within a specific time period. High-burst citations often represent pivotal research that introduces new theories, technical breakthroughs, or resolves controversies. **Figure 5B** shows the top 20 references with the strongest citation bursts, with the paper ‘Autologous Nonmyeloablative Hematopoietic Stem Cell Transplantation in Newly Diagnosed Type 1 Diabetes Mellitus,’ published in JAMA, exhibiting the highest burst intensity of 20.49, indicating that this paper received widespread attention from scholars in the field between 2008 and 2012.

**Table 4:** The top 10 co-cited references of publications in stem cell therapy for diabetes.

Year	First author	Title	Journal	Co-citation	Centrality
1	Carlos E. B. Couri	C-Peptide Levels and Insulin Independence Following Autologous Nonmyeloablative Hematopoietic Stem Cell Transplantation in Newly Diagnosed Type 1 Diabetes Mellitus	JAMA	43	0.02
2	Ju´lio C. Voltarelli	Autologous Nonmyeloablative Hematopoietic Stem Cell Transplantation in Newly Diagnosed Type 1 Diabetes Mellitus	JAMA	42	0.01
3	Felicia W. Pagliuca	Generation of Functional Human Pancreatic β Cells *In Vitro*	Cell	34	0.05
4	Evert Kroon	Pancreatic endoderm derived from human embryonic stem cells generates glucose-responsive insulin-secreting cells *in vivo*	Nature biotechnology	26	0.33
5	Alireza Rezania	Reversal of diabetes with insulin-producing cells derived *in vitro* from human pluripotent stem cells	Nature biotechnology	26	0.03
6	Leonardo Velazco-Cruz	Acquisition of Dynamic Function in Human Stem Cell-Derived b Cells	Stem Cell Reports	25	0
7	Francesca D’Addio	Autologous Nonmyeloablative Hematopoietic Stem Cell Transplantation in New-Onset Type 1 Diabetes: A Multicenter Analysis	Diabetes	24	0.04
8	Pouya Saeedi	Global and regional diabetes prevalence estimates for 2019 and projections for 2030 and 2045: Results from the International Diabetes Federation Diabetes Atlas, 9th edition	Diabetes research and clinical practice	22	0
9	Qiao Zhou	*In vivo* reprogramming of adult pancreatic exocrine cells to b-cells	Nature	21	0.02
10	Gopika G. Nair	Recapitulating endocrine cell clustering in culture promotes maturation of human stem-cell-derived β cells	Nature cell biology	21	0.01

### 3.5 The distribution of journals

**Figure 6:**
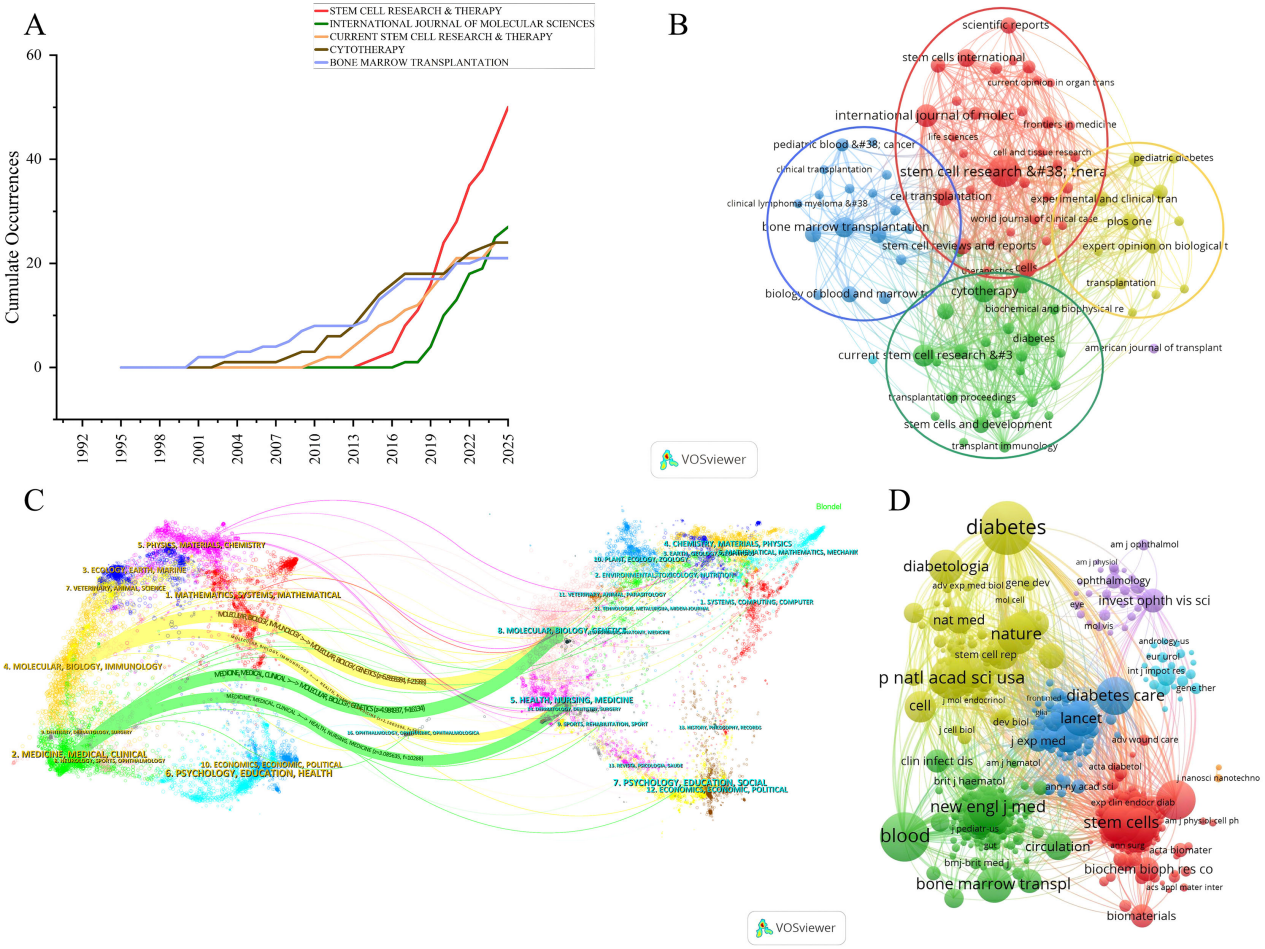
Journals Visualizations. **(A)** Trends in the number of articles published in the top 5 journals over time. **(B)** The visualization map of publishing journals generated based on VOSviewer, with each distinct colored circle representing a different category; **(C)** The dual-map overlay of journals illustrates the citation relationships between citing journals on the left and cited journals on the right. The width of the connecting lines signifies the strength of the citation relationships. **(D)** The visualization map of co-cited journals generated based on VOSviewer.

**Table 5** presents the top 10 journals with the highest number of published papers, representing the active and influential journals in the field of stem cell therapy for diabetes. The journal with the most publications is STEM CELL RESEARCH & THERAPY (50 papers), which also holds the highest impact factor among the top 10 journals, indicating that it is the preferred platform for publishing research in this field. Its leading position highlights the establishment of a stable core knowledge exchange platform in this area. Journals ranked 2nd through 10th have relatively balanced publication numbers, ranging from 27 to 14 papers, forming an important knowledge dissemination network in the field. The distribution of these journals suggests that the application of stem cell therapy in diabetes is a comprehensive frontier field that integrates basic research, clinical translation, and specialized technologies. **Figure 6A** shows the publication trends of the most prolific journals over time, with the trend line indicating a significant increase in publications after 2010, marking the rapid development of the field. Among the top 10 journals, 4 are categorized in the JCR Q1 quartile, and 4 are in the JCR Q2 quartile, indicating that there is a substantial amount of high-quality research in this field. This attracts the attention of top-tier journals, which in turn further demonstrates the academic vitality and influence of the field.

**Table 5:** The top 10 journals in stem cell therapy for diabetes.

Rank	Sources	N(%)	IF^a^ (2024)	JCR^b^ (2024)
1	STEM CELL RESEARCH & THERAPY	50(3.13)	7.30	Q1
2	INTERNATIONAL JOURNAL OF MOLECULAR SCIENCES	27(1.69)	4.90	Q1
3	CURRENT STEM CELL RESEARCH & THERAPY	24(1.50)	2.20	Q4
4	CYTOTHERAPY	24(1.5)	3.20	Q2
5	BONE MARROW TRANSPLANTATION	21(1.31)	5.20	Q1
6	CELL TRANSPLANTATION	17(1.06)	3.20	Q2
7	STEM CELLS INTERNATIONAL	17(1.06)	3.30	Q3
8	STEM CELLS TRANSLATIONAL MEDICINE	17(1.06)	4.90	Q2
9	CELLS	16(1.00)	5.20	Q2
10	BIOLOGY OF BLOOD AND MARROW TRANSPLANTATION	14(0.88)	4.40	Q1

^a^IF: impact factor.

^b^JCR: journal citation reports.

High-output journals typically reflect the research status and hot topics in a given field, while highly cited journals are more commonly considered as the knowledge repositories of the field, indicating broader prospects and development directions. **Table 6** presents the top 10 most-cited journals, which form the core knowledge foundation for stem cell therapy in diabetes. Their high co-citation frequency indicates that the theoretical foundation, research background, and methodologies of many subsequent studies are derived from publications in these journals. The journal with the highest co-citation frequency is Diabetes, with a co-citation count of 3039, highlighting its importance as a key theoretical source in the field of diabetes research. Notably, 9 of the top 10 journals are classified as Q1 journals in the JCR rankings. Among these, Blood (23.10), The New England Journal of Medicine (78.5), Nature (48.5), and Diabetes Care (16.60) have impact factors exceeding 10, and are all top-tier biomedical or general journals with extremely high influence and research value.

**Table 6:** The top 10 co-citation journals in stem cell therapy for diabetes.

Rank	Sources	Co-citations	IF^a^ (2023)	JCR^b^ (2023)
1	DIABETES	3039	7.500	Q1
2	BLOOD	2505	23.10	Q1
3	PROCEEDINGS OF THE NATIONAL ACADEMY OF SCIENCES OF THE UNITED STATES OF AMERICA	1925	9.100	Q1
4	THE NEW ENGLAND JOURNAL OF MEDICINE	1767	78.50	Q1
5	STEM CELLS	1655	3.600	Q2
6	NATURE	1645	48.50	Q1
7	PLOS ONE	1620	2.600	Q2
8	DIABETES CARE	1522	16.60	Q1
9	STEM CELL RESEARCH & THERAPY	1333	7.300	Q1
10	BONE MARROW TRANSPLANTATION	1329	5.200	Q1

^a^IF: impact factor.

^b^JCR: journal citation reports

**Figures 6B, D** respectively display the visual maps of publication journals and co-cited journals generated using VOSviewer. Different colors represent different clusters, and the clustering of journals is based on their interrelations. From **Figure 6B**, it can be seen that the journals in this field form four significant clusters. The red cluster includes journals such as Stem Cells International, Current Opinion in Organ Transplantation, Stem Cell Research and Therapy, and Cell Transplantation, with a common focus on the micro-mechanisms, technical methods, and conceptual validation of stem cell therapy. The yellow cluster, which primarily includes Experimental and Clinical Transplantation, PLOS ONE, Expert Opinion on Biological Therapy, etc., emphasizes reporting on clinical transplantation outcomes and efficacy assessments. The green cluster consists of journals such as Cytotherapy, Diabetes, Current Stem Cell Research & Therapy, Transplantation Proceedings, and Stem Cells and Development, with a greater focus on immune responses following cell therapy and stem cell transplantation. The blue cluster includes journals like Bone Marrow Transplantation, Clinical Lymphoma Myeloma, Clinical Transplantation, and Biology of Blood and Marrow Transplantation, with a focus on hematopoietic stem cell transplantation and its clinical applications.

As shown in **Figure 6D**, the co-cited journals are divided into six major clusters. The yellow cluster, which includes journals such as Nature, Cell, Molecular Cell, Journal of Cell Biology, and Diabetes, mainly focuses on basic cell biology and diabetes-related research. The green cluster, comprising journals like Blood, New England Journal of Medicine, Bone Marrow Transplantation, and Clinical Infectious Diseases, is more focused on influential clinical studies. The red cluster, which includes journals like Stem Cells, Biomaterials, Acta Biomaterialia, and Biochemical and Biophysical Research Communications, is more concerned with basic research in stem cell biology, related biomaterials research, and the basic and clinical studies of stem cell differentiation for treating diabetes and its complications. The blue cluster, consisting of top journals such as The Lancet, Diabetes Care, and Journal of Experimental Medicine, focuses on the mechanistic research and clinical efficacy of stem cell therapy for treating diabetes and its complications. The light blue cluster, including journals like Gene Therapy, International Journal of Impotence Research, and European Urology, focuses more on stem cell therapy for diabetes-related complications, such as erectile dysfunction. The purple cluster, which includes journals like Ophthalmology, American Journal of Ophthalmology, and Investigative Ophthalmology & Visual Science, is also centered on the application of stem cell therapy in diabetic retinopathy, diabetic glaucoma, and other ocular complications.

**Figure 6C** presents a dual-graph overlay of journals in the field, intuitively illustrating the distribution of cited and citing literature, the evolution of citation patterns, and the shifting research focus of these journals. This visualization aids readers in clearly understanding the relationships and dynamics between different disciplines within the application of stem cell therapy to diabetes. The journals on the left represent the forefront of research in the field, while those on the right represent the foundational knowledge in the domain. The colored bands in the middle signify the flow of knowledge from the foundational knowledge base to the research frontier. The thicker the band, the stronger and more significant the knowledge flow from that foundation to the current research frontier. The bands in **Figure 6C** originate from multiple citing journals across disciplines such as molecular biology, immunology, clinical medicine, and pharmacy, suggesting the diverse and highly interdisciplinary nature of the knowledge sources in this field.

**Figure 7:**
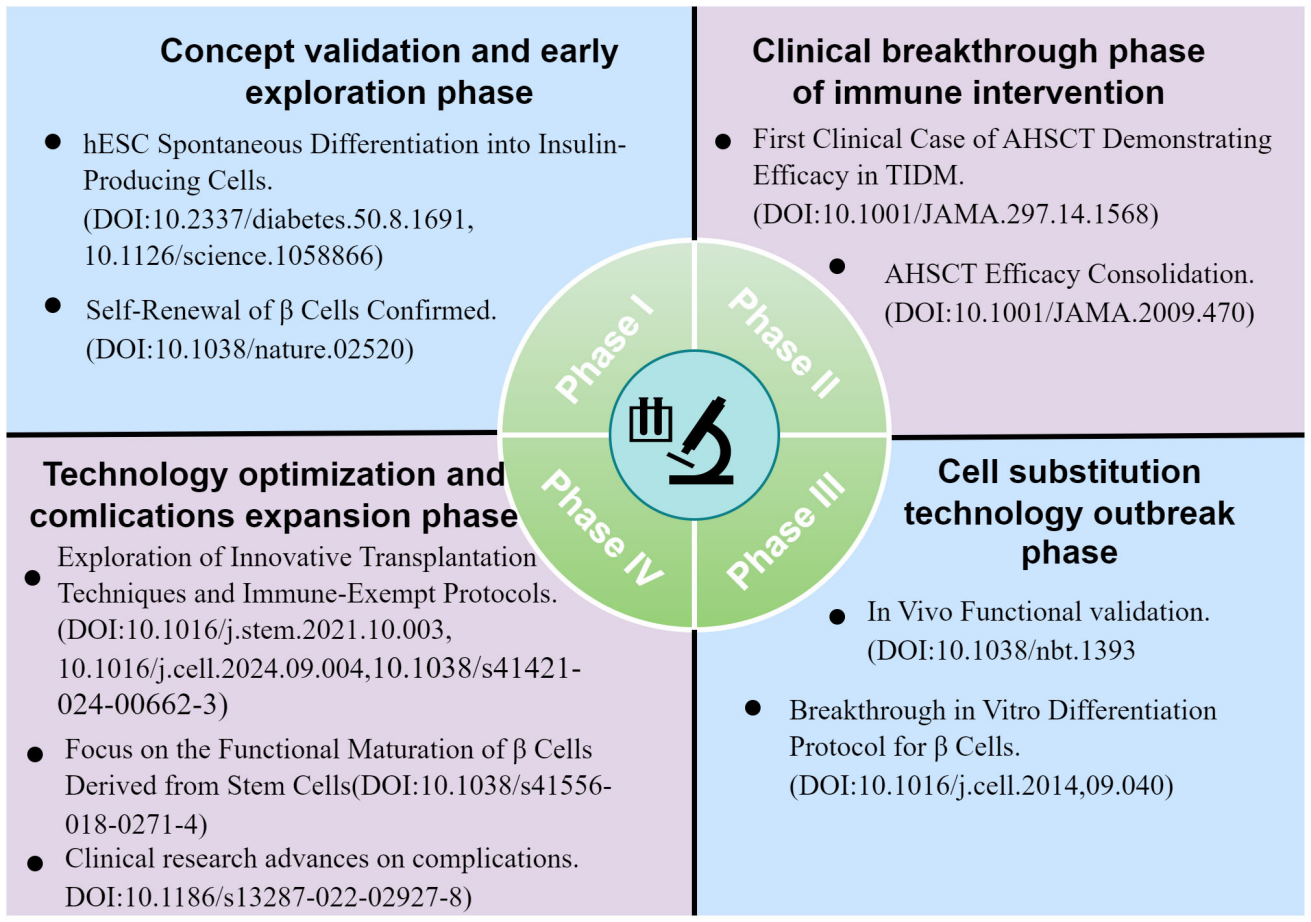
The evolution of stem cell therapy for diabetes. The visual map presents a chronological review of significant scientific achievements in the fields.

### 3.6 A review of significant academic achievements in stem cell therapy for diabetes

The development trajectory of this field is clearly delineated and can be roughly divided into four stages (**Figure 7**): concept validation and early exploration, clinical breakthroughs in immune intervention, technological explosion in cell replacement therapy, and technological optimization with the expansion of complications. In the early stages of development, the core focus of research was on verifying whether stem cell therapy could be used to treat diabetes. Studies by Assady et al. (15) and Lumelsky et al. (16) provided the initial proof of concept, demonstrating that hESCs could spontaneously differentiate into insulin-secreting cells *in vitro*, thus laying the foundation for cell replacement therapy. Subsequently, Dor et al. (17) challenged traditional understanding by proposing that pancreatic β-cells in adult mammals possess self-replication ability, revealing the potential for endogenous regeneration. With advances in science and technology, as well as a deeper understanding, stem cell therapy began to be applied in the clinical treatment of diabetes. A landmark event in this progression was the clinical application of autologous hematopoietic stem cell transplantation (AHSCT). Research teams led by Voltarelli (18) and Couri (19) reported the first clinical study on the use of AHSCT to treat newly diagnosed T1DM in JAMA. This approach involved high-dose immunosuppressive therapy followed by the reinfusion of autologous hematopoietic stem cells, aiming to rebuild the immune system and block autoimmune attacks. The results showed that the majority of patients achieved sustained insulin independence, with a safety profile that was both controllable and acceptable. This experiment demonstrated the clinical feasibility of using immune-reset strategies to treat T1DM, propelling stem cell therapy from the laboratory to clinical practice and sparking global attention in the field. As the immune intervention pathway matured, researchers also began to focus on the directed differentiation of β-cells *in vitro*, which has since seen a series of breakthroughs. As early as 2008, Kroon (20) theoretically demonstrated that the pancreatic endoderm differentiated from hESCs could mature *in vivo* into glucose-responsive endocrine cells. The research teams of Pagliuca (21) and Rezania (22) respectively reported large-scale, efficient protocols for generating functional hESC-derived β-cells in Cell and Nature Biotechnology, and successfully reversed hyperglycemia in diabetic animal models. These milestone studies addressed the core bottleneck of cell replacement therapy—the issue of cell sourcing—by establishing scalable and standardized cell production processes, making the “unlimited supply of β-cells” a reality. As research in stem cell therapies for diabetes deepened, researchers began to shift focus towards host immune responses, cell encapsulation, delivery technologies, and complications of diabetes. Nair et al. (23) focused on strategies such as cell clustering to promote functional maturation of stem cell-derived β-cells, enabling them to match primary adult β-cells in terms of gene expression and secretion dynamics. Research by Ramzy et al. (24)explored gene editing or encapsulation techniques to protect transplanted cells from immune system attacks. The research team of Hongkui Deng at Peking University (25, 26) developed a strategy for subcutaneous implantation in the rectus sheath to transplant hESC-derived pancreatic β-cells into animal models and patients with T1DM, demonstrating good clinical efficacy and providing a new, safe, and effective transplantation option for cell replacement therapy in T1DM. The Yin Hao team at Shanghai Changzheng Hospital has, for the first time in patients with T2DM, utilized autologous stem cell-derived pancreatic islet tissue. This study provides the first clinical evidence demonstrating that stem cell-derived regenerative pancreatic islet tissue can effectively compensate for the loss of pancreatic function in advanced T2DM patients over the long term, reversing hyperglycemia and achieving functional cure (27). Additionally, the research team led by Liu Yi (28) revealed, for the first time, the efficacy and mechanisms of human umbilical cord-derived MSCs in treating diabetic vascular endothelial injury. These findings indicate that stem cell therapy in the field of diabetes has entered a “refined” and “integrated” phase, focused on translating stem cell products into safe, effective, and durable clinical applications.

### 3.7 Keyword analysis

High–frequency keywords reflect the sustained research emphases in a field. Keywords with a centrality ≥0.1 are considered “hub” nodes that connect different research topics and serve as bridges in knowledge flow. Burst keywords (keywords with citation bursts) are identified by detecting a significant increase in their citation or occurrence frequency over a specific period relative to the historical baseline; they are used to recognize research frontiers and emerging topics. A higher burst strength indicates more concentrated attention on the keyword during the burst period. Analysis of the start and end dates of different burst phases allows clear observation of the shifts and evolution of research hotspots. In this study, the interactive interpretation of high–frequency, high–centrality, and high–burst–strength keywords was used to comprehensively identify research hotspots: high–frequency plus high–centrality keywords represent the core structure of the field; high–frequency but low–centrality keywords represent mainstream directions within the field but do not necessarily serve as connectors; keywords with high burst strength but modest total frequency typically represent emerging frontier directions.

The **Table 7** lists the top 20 most frequently occurring keywords in the field of stem cell therapy for diabetes, excluding core keywords such as “stem cell”, “diabetes mellitus”, and “stem cell therapy”. High-frequency keywords like “mesenchymal stem cells”, “bone marrow transplantation”, “differentiation”, “β cell”, and “insulin-producing cell” reflect the most stable, core, and fundamental research elements in this field. Notably, keywords such as “stem cell transplant” and “bone marrow transplant” exhibit the highest centrality, indicating that transplantation technologies are the undisputed core hub of the field. Furthermore, keywords like “therapeutic modalities” and “expression” also show relatively high centrality, suggesting a close connection between research on basic molecular mechanisms and clinical translation.

**Table 7:** The top 20 keywords in stem cell therapy for diabetes.

Rank	Keyword	Occurrences	Centrality	Rank	Keyword	Occurrence	Centrality
1	stem cell	387	0.04	11	type 1 diabetes mellitus	137	0.03
2	diabetes mellitus	288	0.08	12	beta cell	120	0.06
3	mescenchymal stem cells	279	0.02	13	expression	115	0.07
4	stem cell transplant	218	0.12	14	progenitor cell	112	0.05
5	bone marrow	208	0.06	15	*in-vitro*	110	0.05
6	transplantation	184	0.04	16	insulin-producing cell	109	0.01
7	bone marrow transplant	161	0.11	17	mouse	86	0.05
8	stem cell therapy	158	0.03	18	disease	78	0.06
9	differentiation	155	0.03	19	cell therapy	65	0.02
10	therapeutic modalities	147	0.07	20	endothelial progenitor cell	64	0.03

**Figure 8:**
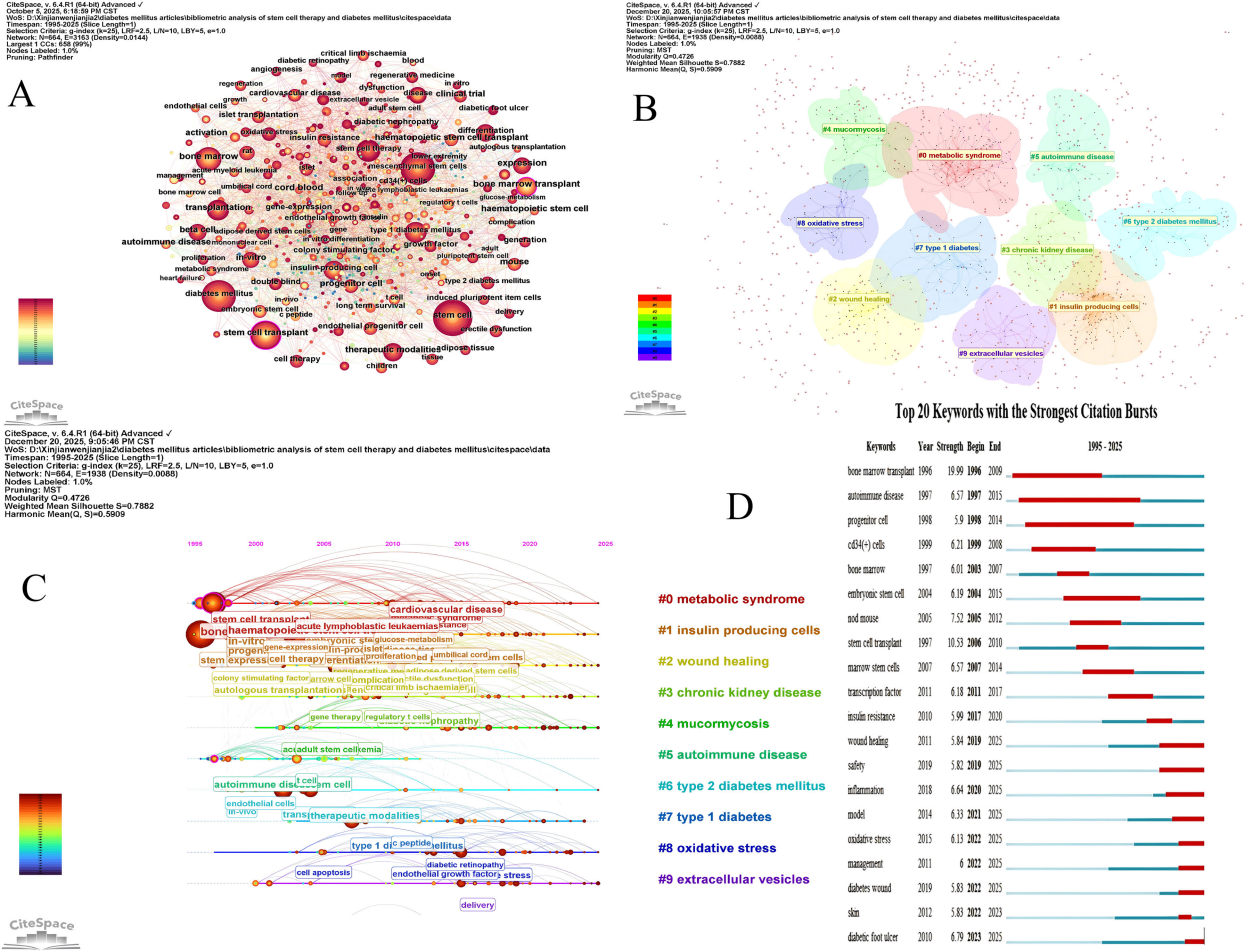
The analysis of keywords related to stem cell therapy for diabetes. **(A)** The keyword co-occurrence map is generated by CiteSpace, where each node represents a keyword. The size of the node indicates the frequency of the keyword, and the thickness of the connections between nodes represents the frequency of co-occurrence of the keywords. **(B)** The keyword clustering map is generated by the LLR algorithm, resulting in 10 clustering labels. Each color cluster represents a different category, with the size of the cluster and the labels revealing the division of subfields. **(C)** The timeline chart related to stem cell therapy for diabetes shows the temporal evolution of the 10 clusters. **(D)** The figure displays the top 20 keywords with the strongest burst, highlighting sudden shifts in information. The red bursts on the timeline represent these abrupt changes. These bursts indicate a rapid increase in keyword frequency and the emergence of important issues or solutions in the field.

**Figure 8A** presents a keyword co-occurrence network generated using CiteSpace. The parameters of CiteSpace were configured as follows: time slice (1995–2025), years per slice (1), term source (entire selection), node type (keyword), and selection criteria (top N = 50), with all other parameters retaining their default settings. Based on this keyword network, we subsequently generated a keyword cluster map, a timeline view, and a keyword burst detection map.

Keyword co–occurrence analysis was used to identify thematic clusters. In CiteSpace, the keyword co–occurrence network was constructed based on the frequency with which two keywords appear together in the same document. After network construction, the LLR algorithm was applied to cluster keywords. Each resulting cluster represents a set of semantically related research topics. Cluster labels are ordered by cluster size in descending order; smaller cluster numbers indicate larger clusters and more central positions in the knowledge network. **Figure 8B** displays 10 distinct colored clusters, representing the top 10 research themes in the field of stem cell therapy and diabetes research: #0 metabolic syndrome, #1 insulin producing cells, #2 wound healing, #3 chronic kidney disease, #4 mucormycosis, #5 autoimmune disease, #6 type 2 diabetes mellitus, #7 type 1 diabetes, #8 oxidative stress, #9 extracellular vesicles. The timeline view illustrates the dynamic evolution of keywords in this field, offering an intuitive representation of shifting research trends and facilitating the identification of emerging research foci. Building on the keyword clustering, we constructed a timeline of keyword clusters (**Figure 8C**), which delineates the research hotspots and trend variations in the application of stem cell therapy for diabetes from a temporal perspective. The keyword burst detection map highlights terms that experience a sudden surge in citation frequency within a short period, reflecting frontier research hotspots and signaling emerging trends. **Figure 8D** displays the top 20 keywords with the highest burst strength in the application of stem cell therapy for diabetes. Analyzing the temporal patterns of these keyword bursts provides a clear overview of the field’s developmental trajectory. Initially, research efforts concentrated on establishing fundamental technical foundations and experimental models. The early burst of “bone marrow transplant” (strength 19.99) underscored the central role of bone marrow/hematopoietic stem cell transplantation as the initial technical cornerstone of the field, with its high burst strength signifying concentrated scholarly attention. The burst of “Nonobese diabetic mice,” a classic mouse model for T1DM, indicated vigorous activity in basic research. Bursts of keywords such as “Cord blood,” “CD34(+),” “embryonic stem cell,” and “*in vitro* differentiation” reflected broad exploration of various stem cell sources and preparation techniques by researchers. This phase was characterized by a focus on methodological development and proof-of-concept studies. The emergence of “C peptide” as a burst keyword marked a pivotal shift, signaling the transition from mere “transplantation” towards precise assessment of β-cell function and therapeutic efficacy. The bursts of keywords like “insulin resistance” and “inflammation” indicated a growing research emphasis on the inflammatory and immune mechanisms underlying type 2 diabetes(T2DM) that stem cell therapies aim to modulate. The bursts of “safety” and “management” suggested the field’s evolution from aggressive efficacy exploration to in-depth investigation of treatment safety, clinical standardization, and long-term patient management. Furthermore, the robust burst of “diabetes wound” unveiled a significant research frontier: utilizing stem cell therapies to treat diabetic complications, particularly refractory diabetic wound healing and diabetic foot ulcers—an area with substantial clinical demand and market potential.

## 4 Discussion

Diabetes is a global health issue characterized by insulin resistance and impaired β-cell function in the pancreas. While traditional treatment methods can temporarily control blood glucose levels, they cannot reverse the progression of the disease. With advancements in medical technology, stem cells have become a research focus in the field of diabetes treatment. This study employs bibliometric analysis to explore the development of stem cell therapy in diabetes research and forecasts future research trends.

### 4.1 An overview of stem cell therapy for diabetes

In this bibliometric analysis, we applied CiteSpace, VOSviewer, and R-bibliometrix to analyze 1,600 research papers related to stem cell therapy and diabetes. The data for these publications were sourced from the Web of Science Core Collection database, covering all available literature in this field up to July 30, 2025. The trend in annual publications shows a continuous upward trajectory in both publication and citation counts, especially from 2010 onward, with a significant surge reaching its peak in 2022. Although there was a slight decline in 2023, we believe this may be attributed to the lag in database indexing. This suggests that the research in this field is currently in the “mature phase” of the scientific evolution cycle, and as scientific inquiry continues to deepen, we anticipate that the volume of publications will continue to rise.

Through statistical analysis of publication volumes by countries/regions and research institutions, we identified the key contributing nations and institutions in this field. The United States and China are the leading countries in terms of publication output, accounting for more than 50% of the total publications in this field. This indicates a clear “bipolar dominance, multipolar follow-up” pattern in the distribution of scientific output, and highlights the unbalanced international distribution of research in this area. Consistent with the trends observed in national distribution, six out of the top 10 institutions by publication volume are based in the United States, and these institutions also exhibit high citation rates for their accumulated literature. The reasons for this may include: a) substantial financial investments in biomedicine in the United States, where high-cost investments facilitate the production of high-quality research outcomes (29); b) the concentration of world-class universities and research institutions, which fosters innovation; c) a powerful biomedical industry that quickly drives clinical translation; d) long-standing leadership in multiple disciplines, with a solid research foundation and interdisciplinary outcomes that have laid a strong academic foundation for stem cell therapy and diabetes research (30); and e) broad academic exchange and cooperation. China ranks second in publication volume, following the United States. The underlying factors may include strong national strategic support, funding investments, substantial patient demand, and a large research workforce. However, it is noteworthy that while China’s publication output is high, the average citation count per paper still needs improvement. Future research should shift from focusing on “quantity” to “quality.” Among the top 10 high-output countries, the United Kingdom, Italy, Germany, and France stand out in terms of citation volume, which is closely linked to their deep academic heritage, longstanding research traditions, and close cooperation within the EU framework (30). Additionally, countries like Iran and Brazil are gradually emerging as key contributors, signaling the field’s diversification and globalization. In terms of collaboration, the United States remains the most closely connected country for international cooperation. However, overall, the field still primarily relies on domestic collaborations, showing a trend of consolidation.

In the author analysis, the most prolific authors in this field are Voltarelli, Julio Cesar, and Bagher Larijani. Among them, Voltarelli, Julio Cesar holds the highest citation count, H-index, and Total Link Strength, indicating his significant influence in the field. Among the top 10 authors by publication volume, Voltarelli, Julio Cesar, Couri, Carlos E B, and three other authors are affiliated with the University of São Paulo, demonstrating a close collaborative relationship. This suggests that the research team centered around Voltarelli, Julio Cesar has a major influence in the field and is a core research force. This team primarily focuses on the potential applications of stem cell therapy and immune intervention in T1DM. While Bagher Larijani has the same publication volume as Voltarelli, Julio Cesar, his citation count does not stand out as much. His research centers on exploring MSCs, particularly those derived from fetal and placental sources, for the treatment of T1DM and its complications (31). His work advances the clinical validation phase by optimizing stem cell delivery methods. Professor Ricordi Chamber from the University of Miami is also a distinguished contributor to the field. His invention of the automated islet separation system has revolutionized the process of efficiently and effectively isolating islets from donor pancreases. This advancement has dramatically improved islet yield and success rates, making large-scale clinical islet transplantation feasible (32).

At the same time, Professor Ricordi Chamber has been actively involved in initiating and participating in several large-scale international clinical trials, focusing on optimizing transplantation protocols and expanding their indications (33–35). Currently, Professor Ricordi Chamber’s team is shifting their focus to the immune tolerance and immune rejection of grafts, exploring the use of biomaterial encapsulation to reduce immune rejection (36). The foundational and clinical research conducted by these investigators has significantly advanced the development of stem cell therapies in the field of diabetes applications.

The top three journals with the highest publication volume in this field are STEM CELL RESEARCH & THERAPY, INTERNATIONAL JOURNAL OF MOLECULAR SCIENCES, and CURRENT STEM CELL RESEARCH & THERAPY, indicating their greater interest in research within this domain. The broad range of publication sources also suggests that stem cell therapies in diabetes research represent an interdisciplinary field that integrates stem cell biology, molecular mechanisms, immunology, and transplantation medicine. The journals cited most frequently represent the foundational sources of research in this field. Diabetes stands out as the leading source, clearly indicating its position as the most fundamental knowledge base. It provides critical insights into the disease’s pathological mechanisms, animal models, and foundational research evidence. In contrast, Diabetes Care, ranked 8th, offers valuable clinical evidence, including clinical management, epidemiology, and treatment endpoints. Journals such as Blood and Bone Marrow Transplantation contribute research evidence on the immunological principles of stem cell therapy and transplantation techniques. Top-tier comprehensive journals like THE NEW ENGLAND JOURNAL OF MEDICINE and NATURE further confirm that high-quality foundational research plays a crucial role as an important theoretical and data source in the fields of stem cell therapy and diabetes research.

By reviewing major scientific achievements in the field and analyzing keyword burst maps, we identified a clear four–stage evolutionary trajectory for stem cell therapy in diabetes. The first stage is concept validation and early exploration. The core feature of this stage was to test the fundamental hypothesis that “stem cells can be used to treat diabetes” *in vitro* and in animal models, focusing on the feasibility of differentiation and initial therapeutic effects. Stem cells were mainly derived from embryonic stem cells, adult stem cells, induced pluripotent stem cells, hematopoietic stem cells, and mesenchymal stem cells (37). These stem cell sources were also incorporated into diabetes research by scholars in the field. Multiple studies successively confirmed that embryonic stem cells, adult stem cells, hematopoietic stem cells, and mesenchymal stem cells could differentiate into insulin–producing cells or islet–like structures *in vitro* or in animal models (15, 38–41). On the other hand, the study by Yuval Dor et al. demonstrated that newly formed β–cells in adult mice after pancreatectomy originate primarily from proliferation of terminally differentiated β–cells, suggesting the potential of activating endogenous repair mechanisms for diabetes treatment (17). Our bibliometric analysis showed that the citation bursts of keywords such as “nonobese diabetic mice”, “hematopoietic stem cell”, “*in vitro* differentiation”, and “embryonic stem cell” during this stage reflected the intensive exploration of differentiation potential at that time. The second stage is clinical breakthrough and immune intervention. The core feature of this stage was the large–scale conduct of clinical trials, marking the leap of stem cell therapy from the laboratory to clinical practice, while autologous hematopoietic stem cell transplantation emerged as a key immune intervention strategy for diabetes. The autologous hematopoietic stem cell transplantation (AHSCT) studies led by teams including Voltarelli and Couri, for the first time, demonstrated the feasibility of treating new–onset T1DM through immune resetting, redefining T1DM as an immune–mediated disease that can be rebooted (18, 19). This breakthrough was reflected bibliometrically by citation bursts around 2010, shifting research emphasis from “transplantation itself” toward precise assessment of β–cell function. In addition, research on type 2 diabetes gradually increased, predominantly using mesenchymal stem cells from various sources and autologous bone marrow stem cell transplantation (42, 43). The extensive clinical trials and immune intervention strategies carried out during the second stage played a crucial bridging role in the development of the field and laid a solid foundation for subsequent in–depth research. The third stage is cell replacement and technological optimization. The landmark achievement of this stage was overcoming the critical bottleneck of cell sourcing. The research teams led by Pagliuca and Rezania successively established protocols for efficiently generating functional β–cells from human pluripotent stem cells and successfully reversed hyperglycemia in diabetic animal models (21, 22). These milestone studies made the “unlimited supply of β–cells” a reality and established the cell replacement paradigm, which is complementary to the immune intervention paradigm. The fourth stage is optimization, integration, and complication expansion. During this period, research focus has shifted toward functional maturation, immune privilege, delivery technologies, and diabetic complications. Representative examples include promoting β–cell maturation through cell clustering (23), protecting grafts by gene editing or encapsulation technologies (24), and developing novel transplantation sites (25, 26). At the same time, evidence has accumulated for the use of mesenchymal stem cells and their exosomes in diabetic foot ulcers, nephropathy, and cardiovascular complications (44–46).

In summary, stem cell therapy for diabetes follows a clear evolutionary logic: from validation of theoretical feasibility, to breakthrough of clinical efficacy, to realization of technological accessibility, and finally toward optimization of therapeutic strategies and diversification of applications — a mature stage.

### 4.2 Identify research hotspots and emerging topics

Keywords represent the core content of a publication, and the evolution of keywords reflects the research hotspots and changing trends within a field. High-frequency keywords indicate the ongoing topics of focus within the field, while keyword clustering encompasses research content related to specific themes. Through keyword co-occurrence and clustering mapping, it is possible to identify the research hotspots and emerging topics in the field of stem cell therapy for diabetes.

Cell replacement therapies, including pancreas and islet transplantation, currently represent the ideal clinical approach for achieving a cure for insulin-deficient/dependent diabetes (2). However, their widespread application is constrained by donor shortage and the immune rejection associated with allogeneic transplantation. Although researchers have attempted to develop ex vivo expansion techniques for primary islet cells and pancreatic tissue, the results have been unsatisfactory (2, 47). Owing to their powerful regenerative and differentiation capacities, stem cells offer a novel solution to overcome the cell–source bottleneck. In recent years, by recapitulating the molecular regulatory processes of pancreatic development *in vivo*, it has become possible to direct the stepwise differentiation of pluripotent stem cells (PSCs) or endodermal stem cells into pancreatic progenitors and even mature insulin–producing β–cells *in vitro* using combinations of cytokines or small molecules (48, 49). Pluripotent stem cells include embryonic stem cells (ESCs) and induced pluripotent stem cells (iPSCs). Early studies confirmed that human ESCs can spontaneously differentiate into insulin–positive cells (15), demonstrating the feasibility of generating β–cells from hESCs. Subsequent research established efficient, reproducible, stage–specific differentiation protocols covering the entire process from definitive endoderm through pancreatic progenitors to insulin–positive cells (50, 51). Currently, mainstream directed differentiation strategies generally follow the steps outlined below. a. Activation of Nodal/Activin and BMP signaling pathways directs PSCs toward definitive endoderm (52), the starting point for all visceral organ development; inhibition of the PI3K (53, 54) or JNK–JUN (55) pathways enhances differentiation efficiency. b. Through regulation of Hedgehog (56, 57), retinoic acid (21, 22), FGF (58, 59), and BMP (60–62) signaling, the endodermal cells are specified into pancreatic progenitors. c. Further induction gives rise to pancreatic endocrine progenitors: activation of NGN3 (a key transcription factor for endocrine differentiation) and inhibition of Notch (21, 22), BMP (63) and TGF–β (61) signaling promote the differentiation of NKX6–1– and SOX9–expressing pancreatic epithelium into endocrine progenitors (64). d. Finally, through modulation of TGF–β (62) and thyroid hormone (65) signaling, the cells are directed to become glucose–responsive, mono–hormonal insulin–expressing β–cells.

However, compared with primary pancreatic β–cells, *in vitro*–derived β–cells are not fully mature at the transcriptional or functional level; they exhibit polyhormonal co–expression and inconsistent expression of mature β–cell marker genes (66, 67), and carry a risk of tumorigenicity (67). To improve differentiation protocols, researchers have also focused on the enrichment of key cell populations and the optimization of culture systems. For example, Mahaddalkar et al. showed that enriching definitive endoderm cells with the surface marker CD177/NBI glycoprotein improves the efficiency of pancreatic differentiation and functional maturation of β–cells (68). Three–dimensional culture systems greatly enhance the expansion and differentiation efficiency of pancreatic progenitors (69). Furthermore, efforts have been made to refine the electrophysiological properties, transcriptional profiles, and metabolic characteristics of islet organoids to bring them closer to adult human islets (11). Despite major breakthroughs in differentiation technology, stem cell–derived β–cells still show low glucose–stimulated insulin secretion and lack the precise regulatory capacity of mature β–cells. Thus, achieving “complete maturation” of *in vitro*–derived β–cells remains a central focus of the field.

Several phase I/II clinical trials of pluripotent stem cell–based islet replacement therapy have achieved important breakthroughs, mostly focusing on type 1 diabetes. ViaCyte was the first company to advance embryonic stem cell–derived pancreatic progenitors encapsulated in a miniature device subcutaneously transplanted into T1D patients, leading to improved blood glucose levels in some patients (24, 70). Vertex adopted a different strategy, transplanting fully differentiated embryonic stem cell–derived islet–like cells (VX–880) into T1D patients (71). In a 2024 clinical trial, all 12 patients receiving the full dose of VX–880 achieved HbA1c levels below 7%, and the latest report indicated that 7 out of 10 participants became insulin–independent after six months (33). Another recent clinical trial successfully transplanted chemically reprogrammed pluripotent stem cell–derived islets under the anterior rectus sheath of a T1D patient, achieving insulin independence within a one–year follow–up (26). These results suggest that transplanting terminally differentiated β–like cells may be a more effective strategy, allowing cell production in a controlled environment and confirmation of critical quality attributes before transplantation, thereby achieving more consistent dosing and better therapeutic outcomes (72). Compared with ESCs, iPSCs avoid ethical and transplantation concerns in clinical application; by 2015, research on human induced pluripotent stem cells had surpassed that on hESCs and has continued to grow annually, while clinical–grade hESC lines for specific diseases have been established and maintained (73). Nevertheless, clinical translation of pluripotent stem cell–based islet replacement therapy for diabetes remains challenging. Because of sufficient space capacity and easier identification of adverse reactions, current clinical trials often use the portal vein and subcutaneous space as transplantation sites (24, 70). However, the subcutaneous site is prone to fibrosis and lacks effective vascularization, leading to massive cell death (74), while portal vein infusion triggers a non–specific inflammatory and thrombotic reaction known as instant blood–mediated inflammatory reaction (IBMIR) due to direct contact with blood, causing significant loss of transplanted islet cells (75). Thus, alternative sites such as the renal capsule, muscle, gastric mucosa, and spleen are being explored (76). Notably, a Chinese team has successfully transplanted iPSC–derived β–like cells under the anterior rectus sheath in rhesus monkeys and diabetic patients, demonstrating a safer and more feasible new approach (25, 26). Allograft–induced immune rejection and recipient autoimmunity compromise transplanted cell survival (77), and long–term immunosuppressive therapy increases the risk of infection and malignancy (78). Therefore, avoiding immune rejection has become a core challenge for the clinical translation of stem cell–based islet replacement therapy. Various strategies have been proposed, including the use of donor–specific autologous cells (26, 27), physical barriers such as microencapsulation to prevent immune rejection (79), and co–transplantation with immunoregulatory cells (79, 80). As islet β–cell differentiation and transplantation technologies mature, avoiding immune rejection appears to be a critical component of cell replacement therapy for diabetes and is increasingly becoming a research hotspot. We believe that with continuous theoretical and technological progress, this challenge will ultimately be overcome.

In addition to cell replacement, another major application strategy of stem cell therapy in diabetes is immune repair, which is primarily achieved by transplanting mesenchymal stem cells (MSCs) to modulate immunity, protect pancreatic β–cells, and improve insulin resistance (10, 81). MSCs are adult stem cells with self–renewal and multi–lineage differentiation potential, and they can be obtained from various tissues, including bone marrow, adipose tissue, and umbilical cord. They exhibit low immunogenicity because they do not express major histocompatibility complex class II (MHC–II) on their surface (82, 83). At the same time, they express adhesion molecules such as C–C motif chemokine receptor type 2 (CCR2) and C–X–C motif chemokine receptor type 4 (CXCR4), which enable specific migration to inflammatory sites. These two fundamental properties are closely related to the immune–repair potential of MSCs (83).

T1DM is characterized by autoimmune destruction of pancreatic β–cells, whereas T2DM is characterized by chronic low–grade systemic inflammation, and immune cell infiltration as well as inflammatory responses are major drivers of insulin resistance and β–cell dysfunction (84). MSCs possess potent immunomodulatory capacity. On one hand, they can directly inhibit the abnormal proliferation and excessive activation of T cells and B cells through cell–to–cell contact, and upregulate dendritic cells that induce immune tolerance (85). At the same time, MSCs express PD–L1, which binds to PD–1 on T cells, inducing T–cell apoptosis and driving them into a quiescent state (86). On the other hand, MSCs regulate the differentiation and polarization of immune cell subsets, promote the proliferation of regulatory T cells (Tregs), indirectly suppress effector T cells, or participate in immune regulation through paracrine signaling (87). As a result, MSCs have attracted widespread attention for the treatment of both type 1 and type 2 diabetes.

Numerous clinical trials have demonstrated the efficacy and safety of MSC therapy for diabetes. A randomized double–blind, placebo–controlled phase I/II trial showed that after umbilical cord–derived MSC infusion, the decline in fasting C–peptide in type 1 diabetes patients was significantly lower than in the placebo group, and insulin requirements did not increase significantly during the 12–month follow–up (88). A Chinese phase II randomized double–blind, placebo–controlled trial also demonstrated that UC–MSCs significantly improved HbA1c levels and reduced insulin requirements in patients with type 2 diabetes (89). Notably, recent clinical trials have begun to incorporate immunological parameters into their evaluation systems, explicitly using inflammatory markers such as IL–6, IL–10, CRP, and D–dimer as secondary endpoints to assess the efficacy of MSC therapy as an adjunctive treatment for T1DM (90).

The immunomodulatory effects of MSCs are specifically reflected in their ability to inhibit the proliferation and activation of autoreactive T cells, thereby reducing the autoimmune attack on pancreatic β–cells in type 1 diabetes (91). MSCs also decrease Th1 and Th17 subsets, increase Th2 and Treg subsets, and enhance anti–inflammatory cytokine secretion, effectively correcting T–cell subset imbalance (92). Moreover, MSCs enhance the body’s anti–inflammatory capacity by regulating dendritic cell secretion of TNF–α and IL–10, and influence natural killer cell proliferation, cytokine secretion, and cytotoxicity (93). Interleukin–10 (IL–10), a potent anti–inflammatory cytokine, induces macrophage polarization toward the M2 phenotype (94). Multiple studies have shown that IL–10 levels are decreased in obese patients with insulin resistance (95), whereas repeated UC–MSC infusions increase IL–10 levels in insulin–resistant obese mouse models, promote M2 polarization, and alleviate insulin resistance (96). NLRP3 inflammasome activation is closely associated with type 2 diabetes and insulin resistance; high glucose concentrations activate the NLRP3 inflammasome in mouse pancreatic islet cells, inducing IL–1β production and causing insulin resistance (97). In contrast, intravenous injection of UC–MSCs blocks NLRP3 inflammasome activation, inhibits the release of inflammatory factors, and effectively alleviates palmitate– and lipopolysaccharide–induced insulin resistance (98). Through a systematic literature search, we identified representative clinical and preclinical studies that directly reported immunological parameters. **Table 8** summarizes the key findings of these studies, including the types of MSCs used, disease models, and the principal immunological outcomes. This compilation complements the bibliometric data by illustrating how MSCs modulate the immune system to preserve β–cell function, improve insulin sensitivity, and alleviate diabetic complications.

**Table 8:** Key immunological findings from representative clinical and preclinical studies of stem cell therapy for diabetes.

Category	Title	Model	Cell type	Key immunological findings
Clinical trial	Mesenchymal stem cell transplantation in newly diagnosed type-1 diabetes patients: a phase I/II randomized placebo-controlled clinical trial	Newly diagnosed T1DM patients	Autologous bone marrow-derived MSCs	Shifted serum cytokine pattern from pro-inflammatory to anti-inflammatory. Increased the number of regulatory T cells (Tregs) in peripheral blood.
Clinical trial	Safety and preliminary efficacy of mesenchymal stromal cell (ORBCEL-M) therapy in diabetic kidney disease: a randomized clinical trial (NEPHSTROM)	Adults with T2D and progressive diabetic kidney disease	Allogeneic bone marrow-derived, anti-CD362-selected MSCs	Preserved circulating Tregs and reduced natural killer T (NKT) cells in MSC-treated patients. Stabilized inflammatory monocyte subsets, indicating reduced chronic inflammation. No anti-HLA antibody induction in most recipients, confirming low immunogenicity and good safety.
Clinical trial	Preserved beta-cell function in type 1 diabetes by mesenchymal stromal cells	T1DM patients	Autologous bone marrow-derived MSCs	Inhibited T-cell proliferation. Increased regulatory T cell frequency. Reduced production of pro-inflammatory cytokines.
Clinical trial	Adipose tissue-derived stromal/stem cells + cholecalciferol: a pilot study in recent-onset type 1 diabetes patients	Recent-onset T1DM patients	Allogeneic adipose-derived MSCs	Increased FoxP3^+^ Tregs. Inverse correlation between FoxP3 expression and IDAA1c provided direct clinical evidence linking Treg upregulation to improved metabolic outcomes.
Clinical trial	Allogeneic mesenchymal precursor cells (MPC) in diabetic nephropathy: a randomized, placebo-controlled, dose escalation study	T2DM patients with diabetic nephropathy	Allogeneic bone marrow-derived mesenchymal precursor cells (rexlemestrocel-L)	No induction of donor-specific anti-HLA antibodies (low immunogenicity). Patients with higher baseline IL-6 levels showed greater improvement in GFR, suggesting that inflammatory status may influence treatment efficacy.
Clinical trial	Efficacy and safety of umbilical cord-derived mesenchymal stem cells in Chinese adults with type 2 diabetes: a single-center, double-blinded, randomized, placebo-controlled phase II trial	Chinese adults with T2DM	Umbilical cord-derived MSCs	Improved glucose infusion rate (GIR) measured by hyperinsulinemic-euglycemic clamp, indicating enhanced peripheral insulin sensitivity – an effect linked to reduced chronic low-grade inflammation. No improvement in β-cell function (HOMA-β), confirming the primary effect on insulin resistance.
Preclinical study	Mesenchymal stem cells from mouse hair follicles inhibit the development of type 1 diabetes	STZ-induced T1DM mouse model	Mouse hair follicle-derived MSCs (moMSCORS)	Inhibited CD4^+^ T cell proliferation and activation. Reduced M1 pro-inflammatory macrophages and Th17 cells. Increased regulatory T cells (Tregs). Decreased insulitis and preserved insulin secretion, thereby reducing T1DM incidence.
Preclinical study	The impact of human umbilical cord-derived mesenchymal stem cells on the pancreatic function of type 2 diabetic mice and their regulatory role on NLRP3 inflammasomes	HFD + low-dose STZ-induced T2DM mouse model	Human umbilical cord-derived MSCs	Reduced pancreatic inflammation by inhibiting macrophage NLRP3 inflammasome activity. Enhanced autophagy. Preserved islet structure and function.
Preclinical study	The bone mesenchymal stem cell-derived exosomal miR-146a-5p promotes diabetic wound healing in mice via macrophage M1/M2 polarization	Full-thickness skin wound model in diabetic mice; high-glucose-cultured HUVECs and macrophages	BMSC-derived exosomes (EXO-miR-146a-5p)	Promoted macrophage polarization from M1 (pro-inflammatory) to M2 (anti-inflammatory/repair) phenotype. Suppressed TRAF6 expression, enhancing endothelial cell proliferation, migration, and angiogenesis. Accelerated wound healing.
Preclinical study	Exosomal miR-25 from mesenchymal stem cells inhibits T cell migration and alleviates type 1 diabetes mellitus by targeting CXCR3	T1DM mouse model (NOD mice)	MSC-derived exosomes	Delivered miR-25 to activated T cells, down-regulating the chemokine receptor CXCR3. Reduced T cell migration to the pancreas, attenuating autoimmune attack. Preserved islet function.
Preclinical study	Amelioration of diabetic nephropathy in mice by a single intravenous injection of human mesenchymal stromal cells at early and later disease stages is associated with restoration of autophagy	STZ-induced diabetic nephropathy mouse model	Human umbilical cord-derived MSCs	Early injection (8 weeks) significantly reduced serum IL-6, TNF-α, and TGF-β1. Late injection (16 weeks) reduced TGF-β1 only. Restored intra-renal autophagy (reduced p-mTOR/mTOR, p62; increased ULK1, Atg12, LC3B/LC3A). Renoprotective effects independent of blood glucose control, indicating direct immunomodulatory and anti-fibrotic actions.

It should be noted that the immunomodulatory capacity of MSCs is environment–dependent. When exposed to high concentrations of pro–inflammatory cytokines (e.g., TNF–α, IFN–γ) or when Toll–like receptor 3 is activated by its ligand, UC–MSCs exert immunosuppressive effects by inhibiting T–cell proliferation and activation, inducing Treg expansion, and promoting macrophage polarization toward the anti–inflammatory M2 phenotype (84). Conversely, under conditions of low inflammatory cytokine expression or during the early phase of inflammation, UC–MSCs secrete chemokines that recruit lymphocytes, leading to pro–inflammatory effects (99). Furthermore, under hypoxic conditions, MSCs exhibit enhanced proliferation and migration, increased expression of immunomodulatory factors such as human leukocyte antigen G, prostaglandin E2, and indoleamine 2,3–dioxygenase, and reduced apoptosis (99).

Accumulating evidence indicates that β–cell dedifferentiation is a major cause of T2DM, and MSCs can reverse part of this dedifferentiation (100, 101). This effect is mediated by the secretion of IL–1 receptor antagonist (IL–1Ra) by MSCs in response to the strong inflammatory environment of T2DM (102). IL–1Ra inhibits the IL–1 signaling pathway, prevents damage caused by inflammatory intermediates such as COX–2 (103) and NF–κB (104), reduces endogenous IL–1β production in T2DM islets (105), and modulates macrophage phenotype switching (106). These findings further confirm the great potential of MSCs in improving pancreatic β–cell function.

Paracrine signaling is one of the main mechanisms by which MSCs exert their therapeutic effects, and the exosomes secreted by MSCs (MSC–Exos) have been shown to play important roles in the development and progression of various diseases. Exosomes are cup–shaped, lipid bilayer–enveloped nanoparticles. MSC–derived exosomes (MSC–Exos) can be isolated from immortalized MSCs and retain the homing property of MSCs to injured tissues. Through maintaining and recruiting endogenous stem cells, inhibiting apoptosis, modulating immunity, and stimulating angiogenesis, MSC–Exos hold great potential for tissue repair and regeneration (107, 108). Immunomodulation and anti–inflammation are central mechanisms by which MSC–Exos exert their therapeutic effects. The miRNAs they carry, such as miR–216a–5p and miR–146a–5p, can suppress inflammatory pathways including TLR/NF–κB/PI3K–Akt, regulate macrophage polarization, significantly reduce pro–inflammatory cytokines, and increase IL–10 and TGF–β (109). MSC–Exos also modulate the balance between Th1 and Th2 cells, restore Th1/Th2 homeostasis, and promote Treg differentiation (110, 111). Moreover, MSC–Exos have been shown to inhibit natural killer (NK) cell proliferation and activation and reduce their cytotoxicity by mediating TGF–β/Smad signaling (112). Relevant studies indicate that MSC–Exos can suppress pancreatic islet inflammation, prevent excessive immune activation and autoimmune damage, and effectively delay the onset and progression of type 1 diabetes (113, 114). Compared with MSCs, MSC–Exos have lower immunogenicity, no tumorigenicity, greater safety, and lower ethical risks (115). Currently, sources for MSC–Exos used in T1DM therapy include bone marrow (116), adipose tissue (113), human umbilical cord (117), and menstrual blood (117). Among these, umbilical cord–derived MSC–Exos are the most widely used. Studies have shown that intravenous administration of UC–MSC–Exos alleviates hyperglycemia and peripheral insulin resistance in T2DM mice, enhances hepatic glycogen storage, inhibits pancreatic β–cell apoptosis, and promotes muscle glucose metabolism, thereby increasing insulin secretion (118). Further research has revealed that UC–MSC–Exos treatment improves intestinal barrier integrity in T2DM mice, restores immune balance in the liver and adipose tissue by reducing macrophage infiltration and pro–inflammatory cytokine expression, and alleviates lipotoxicity–induced endoplasmic reticulum stress, thereby reducing lipid accumulation and chronic inflammation in the liver and adipose tissue (119, 120). Menstrual blood–derived MSC–Exos have been shown to increase β–cell mass and insulin production in the pancreas of STZ–induced diabetic animal models, and the mechanism involves pancreatic islet regeneration through a PDX–1–dependent pathway (121). Moreover, evidence suggests that BMMSC–Exos may be safer, faster, easier to inject, and more effective than BMMSCs themselves (116). These findings indicate that MSCs can target and enhance the activity of specific enzymes, thereby precisely activating the expression of certain receptor proteins and other physiologically active substances, alleviating and delaying the progression of diabetes. As a nanomedicine, the efficacy of exosomes must also consider the impact of physical barriers. During targeting to tissues and cellular uptake, exosomes need to evade hepatic and renal clearance, avoid binding to serum proteins, escape immune system surveillance, and interact with the vascular endothelium and extracellular matrix. Even if they successfully overcome these barriers and reach the target tissue, they must still avoid lysosomal degradation to function properly (122). Fortunately, their unique cup–shaped structure and lipid bilayer membrane protect their cargo from degradation by the physical environment and provide a long–lasting release effect (92). This means that exosomes have distinct advantages in the treatment of diabetes and may become potential “smart” nanomedicines for cell–free therapy. However, exosomes are highly heterogeneous assemblies of multiple bioactive molecules, and their therapeutic effects likely arise from synergistic actions rather than any single component. Therefore, identifying and quantifying the active or functionally optimized constituents within exosomes—thereby enabling tailored exosome-based therapies or developing exosome editing strategies—would help improve treatment safety and efficacy (123, 124). Furthermore, establishing standardized protocols for exosome culture, extraction, isolation, and characterization to ensure high–quality production and compositional purity, as well as employing preconditioning, genetic engineering, and biomaterial integration to optimize the function of exosomes in diabetic wound healing, remain key technological breakthroughs to enhance therapeutic efficiency and represent a major future research focus.

Stem cell therapy has achieved remarkable success in both basic research and clinical trials for diabetes. As the field matures, the research frontier is expanding from treating diabetes per se to managing its severe complications. Vascular endothelial injury induced by diabetes is a central driver of the development and progression of various diabetic vascular complications. Diabetic nephropathy, one of the most severe complications, is a leading cause of end–stage renal disease. Mesenchymal stem cells (MSCs) have shown great potential in treating diabetic nephropathy. Zhang et al. (45) found that UC–MSCs inhibit epithelial–mesenchymal transition (EMT) and renal fibrosis by delivering exosomes and targeting Hedgehog/SMO signaling. Moreover, UC–MSC–derived miR–342–3p and miR–146a–5p inhibit pyroptosis of renal tubular epithelial cells (125) and promote M2 macrophage polarization by targeting the NLRP3/Caspase1 pathway and TRAF6, respectively. These findings provide a novel intervention strategy for miRNA–modified cell–based therapy of diabetic nephropathy. Endothelial progenitor cells (EPCs), a type of vascular endothelial stem cell, have demonstrated favorable efficacy in promoting angiogenesis and vascular repair in diabetes. Injection of EPCs isolated from human peripheral blood into diabetic mice with hindlimb ischemia promoted EPC homing to the ischemic area, increased blood flow in the ischemic limb, accelerated wound healing, and elevated vascular endothelial growth factor (VEGF) levels (44). Bone marrow–derived MSCs (BMMSCs) have also been shown to significantly ameliorate symptoms of diabetic peripheral vascular disease and reduce the amputation rate, with wound healing speed positively correlated with BMMSC concentration (126). In diabetic cardiomyopathy, BMMSCs reduced myocardial fibrosis and hypertrophy, induced myocardial and vascular regeneration, and improved cardiac remodeling by enhancing matrix metalloproteinase–2 (MMP–2) activity and reducing MMP–9 mRNA transcription (46). For diabetic retinopathy, MSC therapy increases the number of retinal ganglion cells and effectively prevents early retinal vascular damage and retinal thickening, thereby reducing retinal structural damage induced by diabetes mellitus (127). Regarding nerve injury, MSCs can home to the brain microenvironment in stroke rats and restore neurological function, and hypoxia–preconditioned MSCs exhibit enhanced neuronal regeneration properties, thus improving diabetic neuropathy (128). In wound healing, adipose–derived mesenchymal stem cells (ADSCs) have great potential for diabetic wound repair due to their wide availability and their ability to secrete multiple cytokines involved in wound healing (129). ADSC–derived exosomes (ADSC–exos) promote macrophage polarization toward the M2 phenotype, enhance fibroblast proliferation and migration, and improve the angiogenic capacity of vascular endothelial cells by regulating immune responses (130, 131). Furthermore, ADSC–exos alleviate the functional inhibition of keratinocytes, endothelial cells, human skin fibroblasts, endothelial progenitor cells, and human umbilical vein endothelial cells under high–glucose conditions (132).

### 4.3 Current challenges and future prospects

Stem cell therapy represents a critical frontier in modern medicine, advancing at an unprecedented rate. Despite its considerable therapeutic potential, the clinical translation of stem cell therapies faces numerous pressing scientific and technological hurdles that must be overcome: 1) Challenges in Cell Product Quality and Standardization: a. The β-like cells derived from the directed differentiation of hPSCs exhibit transcriptomic and functional profiles that are more akin to fetal or immature β cells. Their glucose-stimulated insulin secretion, particularly in the first-phase response, remains inferior to that of primary adult human pancreatic β cells (21, 133). b. Differentiation protocols currently do not achieve 100% purity, and the final products may contain residual undifferentiated pluripotent stem cells or other pancreatic endocrine cell types. The presence of undifferentiated pluripotent stem cells poses a tumorigenic risk, while the inclusion of other endocrine cell types may compromise the precision and safety of the therapy (134, 135). c. Clinical-grade stem cell products necessitate standardized, reproducible, and scalable manufacturing processes, encompassing all stages from donor qualification to product release (72). Immune rejection remains a persistent challenge for the clinical application of stem cell therapy in diabetes. Even for mesenchymal stem cells, which are considered to have low immunogenicity, their long–term survival and functional maintenance after transplantation are constrained by the host immune environment (136). Meanwhile, long–term use of immunosuppressive agents increases the risks of infection and transplantation–related mortality (137). 3) Although several clinical trials have demonstrated the preliminary safety and efficacy of stem cell–based therapies, most of these trials have relatively short follow–up periods. Consequently, the long–term survival of transplanted cells, their functional persistence, and potential adverse effects remain inadequately evaluated. In particular, there is a tumorigenic risk associated with stem cell therapy. The extensive expansion of stem cells through serial passages and prolonged *in vitro* culture before clinical application can lead to telomere length alterations and chromosomal instability, increasing the risk of genetic mutations and malignant transformation (138). Thus, more extensive and longer–term follow–up studies are needed in the future. 4) Regulatory and ethical issues also pose significant challenges for the clinical translation of stem cell therapies. The use of embryonic stem cells remains ethically controversial, and although induced pluripotent stem cells circumvent the issue of embryonic origin, the safety of the genetic manipulation and reprogramming processes still requires further validation. Moreover, regulatory policies for stem cell–based therapies vary across different countries and regions, and standardized, reproducible cell manufacturing and quality control systems have not yet been fully established. This hampers the conduct of multi–center clinical trials and the widespread application of these therapies, and also poses challenges for international collaboration and the implementation of clinical trials.

Based on this, we anticipate that future research in this field will trend toward more refined cell engineering. A key area of focus will be overcoming the challenge of β-cell maturation and differentiation, which necessitates a deeper understanding of the molecular pathways underlying β-cell maturation both *in vivo* and *in vitro*. Additionally, the development of immune evasion technologies will become a central focus of research. This includes the creation of novel biocompatible encapsulation materials and the exploration of local or short-term immune modulation strategies, such as co-transplantation with regulatory T cells or the use of targeted immunosuppressive agents as alternatives to current broad-spectrum, long-term immunosuppressive therapies. Such strategies aim to achieve a balance between protecting the graft and maintaining the host’s immune function. Furthermore, the immune-regulatory and regenerative capabilities of MSCs and their exosomes, along with other acellular products, show promise for treating diabetes complications. These therapies typically offer higher safety, lower immunogenicity, and greater potential for clinical translation.

In summary, stem cell therapy for diabetes is at a critical juncture, transitioning from proof-of-concept studies to potential clinical solutions. Our bibliometric analysis reveals two core paradigms driving the development of this field: cell replacement — the generation of functional β–cells from pluripotent stem cells through directed differentiation — and immunomodulation — involving the regulation of T–cell subsets, macrophage polarization, and cytokine networks. Future breakthroughs will not solely depend on advances in cell biology but will require an integration of materials science, immunology, genetic engineering, and clinical medicine. By addressing fundamental challenges related to cell function, immune rejection, and long-term safety, the next generation of stem cell therapies may provide safer, more effective, and more universally applicable clinical strategies for achieving functional cures for diabetes.

### 4.4 Limitations

This study has certain limitations. First, due to the software’s adaptability, the research only included literature from the Web of Science Core Collection database. While it is an authoritative database for scientometric analysis, it inevitably introduces selection bias, potentially overlooking high-quality studies published in non-English languages, regional journals, or conference proceedings. Second, bibliometrics primarily analyzes the external characteristics of literature, which is a macro, associative quantitative analysis, rather than a qualitative evaluation of the depth of research content. Additionally, keyword co-occurrence analysis depends on the keywords provided by the authors, and the standardization and consistency of their expressions directly influence the accuracy of clustering results, which may introduce semantic gaps. Moreover, the knowledge map constructed in this study presents a relatively static macrostructure. Although citation burst analysis and the identification of key academic achievements in the field help to provide a dynamic perspective, it remains challenging to fully capture the subtle and complex flow of knowledge and the evolving dynamics within the field. Furthermore, overrepresentation of highly cited countries and institutions is an inherent methodological bias in bibliometric analyses. In this study, the United States and China together account for more than 50% of the total publications, and six of the top ten institutions are located in the United States. While this concentration may partially reflect the actual research investment, academic traditions, and English–language advantages of these countries, it may also lead to a systematic underestimation of high–quality research from other countries or regions. A similar concentration bias may also apply to highly cited papers and core authors. Lastly, the rapid development of technical terminology in stem cell research, combined with the frequent use of synonyms and near-synonyms, presents challenges. Despite efforts in data cleaning, it remains difficult to eliminate potential noise caused by terminology inconsistencies. The high level of interdisciplinary overlap in the field further complicates the precise delineation of its boundaries. Although rigorous search strategies were employed to mitigate this issue, some important literature from the interdisciplinary intersection may have been missed. Future research will adopt cross-validation using multiple databases to enhance the comprehensiveness of the data. Additionally, natural language processing and text mining techniques will be used for deeper content analysis of abstracts and full texts, with the aim of improving the completeness and richness of the field’s knowledge map.

## 5 Conclusion

This study, using CiteSpace, VOSviewer, and the “bibliometrix” R package, conducts a comprehensive analysis of the distribution of countries/regions, institutions, and authors in the stem cell therapy and diabetes research fields from 1995 to 2025. By reviewing major academic contributions, analyzing highly co-cited literature, and identifying research hotspots, the study outlines the current state of the field and predicts future trends and developments. Our research enhances understanding of the field and offers new insights and approaches for the use of stem cells in diabetes treatment.

## Data Availability

The original contributions presented in the study are included in the article/supplementary material. Further inquiries can be directed to the corresponding author.
